# Differential expression of porcine microRNAs in African swine fever virus infected pigs: a proof-of-concept study

**DOI:** 10.1186/s12985-017-0864-8

**Published:** 2017-10-17

**Authors:** Fernando Núñez-Hernández, Lester Josué Pérez, Marta Muñoz, Gonzalo Vera, Francesc Accensi, Armand Sánchez, Fernando Rodríguez, José I. Núñez

**Affiliations:** 1 IRTA, Centre de Recerca en Sanitat Animal (CReSA, IRTA-UAB), Bellaterra, Spain; 2 Centro Nacional de Sanidad Agropecuaria (CENSA), La Habana, Cuba; 3 Departament de Genètica Animal, Centre de Recerca en AgriGenòmica (CRAG), CSIC-IRTA-UAB-UB, Universitat Autònoma de Barcelona, Bellaterra, Barcelona, Spain; 4 Departament de Sanitat i Anatomia Animals, Facultat de Veterinària, UAB, Bellaterra, 08193 Barcelona, Spain; 5 Departament de Ciència Animal i dels Aliments, Universitat Autònoma de Barcelona (UAB), Bellaterra, Barcelona, Spain

## Abstract

**Background:**

African swine fever (ASF) is a re-expanding devastating viral disease currently threatening the pig industry worldwide. MicroRNAs are a class of 17–25 nucleotide non- coding RNAs that have been shown to have critical functions in a wide variety of biological processes, such as cell differentiation, cell cycle regulation, carcinogenesis, apoptosis, regulation of immunity as well as in viral infections by cleavage or translational repression of mRNAs. Nevertheless, there is no information about miRNA expression in an ASFV infection.

**Methods:**

In this proof-of-concept study, we have analyzed miRNAs expressed in spleen and submandibular lymph node of experimentally infected pigs with a virulent (E75) or its derived attenuated (E75CV1) ASFV strain, as well as, at different times post-infection with the virulent strain, by high throughput sequencing of small RNA libraries.

**Results:**

Spleen presented a more differential expression pattern than lymph nodes in an ASFV infection. Of the most abundant miRNAs, 12 were differentially expressed in both tissues at two different times in infected animals with the virulent strain. Of these, miR-451, miR-145-5p, miR-181a and miR-122 presented up-regulation at late times post-infection while miR-92a, miR-23a, miR-92b-3p, miR-126-5p, miR-126-3p, miR-30d, miR-23b and miR-92c showed down-regulation. Of the 8 differentially expressed miRNAs identified at the same time post-infection in infected animals with the virulent strain compared with animals infected with its attenuated strain, miR-126-5p, miR-92c, miR-92a, miR-30e-5p and miR-500a-5p presented up-regulation whereas miR-125b, miR-451 and miR-125a were down-regulated. All these miRNAs have been shown to be associated with cellular genes involved in pathways related to the immune response, virus-host interactions as well as with several viral genes.

**Conclusion:**

The study of miRNA expression will contribute to a better understanding of African swine fever virus pathogenesis, essential in the development of any disease control strategy.

**Electronic supplementary material:**

The online version of this article (10.1186/s12985-017-0864-8) contains supplementary material, which is available to authorized users.

## Background

African swine fever (ASF) is an infectious disease affecting domestic and wild pigs caused by African swine fever virus (ASFV) belonging to the *Asfarviridae* family. ASFV is an icosahedral, enveloped, dsDNA virus ranging from 170 to 193 kb [1]. ASF genomes contain between 150 and 175 ORFs with 34 of them encoding for structural viral proteins and so far, half of them do not have any known function [2]. Sixteen different genomes have been fully sequenced corresponding to virulent, attenuated and non-pathogenic ASFV strains isolated from pigs, wild boars, ticks and tissue culture cells. So far, 22 genotypes have been identified with several of them currently co-circulating in Sub-Saharan Africa. ASF was first described in 1921 in Kenya [3] spreading rapidly throughout other African countries. The disease is endemic in most of Sub-Saharan Africa and in Sardinia [4]. ASFV entered Europe for the first time in the early fifties remaining on the continent until its eradication from Spain and Portugal in 1995 [5]. In 2007 the virus re-entered Europe through Georgia and since then it has spread to East-Europe, becoming a real threat for all Europe. Confirming the menace, in 2014 four countries from the EU declared ASF outbreaks [6]. From 2014 to date, the presence of the disease and more outbreaks have been reported by the World Organization for Animal Health (OIE) in Estonia, Latvia, Lithuania, Poland, Russia, Belarus, Moldova and Ukraine while recently (June 2017), Czech Republic has reported its first case of ASFV [7].

ASFV transmission is produced by direct contact between infected and susceptible animals and alternatively, following indirect transmission routes through contaminated pork, fomites, vehicles, people and ticks. The disease is characterized by an acute form that leads to fever, hemorrhages and several lesions causing death in a short period of time. High mortality leads to huge economical losses in affected areas [7, 8]. Experimental infections occur with the development of clinical signs at 7–10 days post inoculation that can be considered late times of infection. At that time, histopathological evaluation shows typical alterations of the disease such as macrophage infiltrations and apoptotic cells together with a cytokine storm characteristic of the terminal phase in ASF infections according to previous studies [9].

No commercial vaccine is available in spite of the efforts carried out to achieve it. Engineered live attenuated strains and cell culture adapted virus have been the two most common approaches used to obtain a candidate vaccine [10, 11]. Cell culture adaptation leads to attenuation that generally ends rendering non-pathogenic viruses invalid for vaccine purposes. The genomic changes responsible of the phenotypic modification and the mechanisms involved are not well understood [12].

MicroRNAs (miRNAs) are small RNA molecules that can regulate gene expression post-transcriptionally. miRNAs derive from the primary miRNA (pri-miRNA) [13], which is transcribed in the nucleus and processed into a precursor (pre-miRNAs) by the endonuclease Drosha and DCGR8 [14]. The pre-miRNA molecule is transported to the cytoplasm by exportin-5 [15, 16]. Another endonuclease, Dicer [17], processes the pre-miRNA to generate the double-stranded 18–23 nt miRNA duplex. The mature miRNA strand in which the first 2–8 nucleotides (seed region) are essential for recognizing the target mRNA, is incorporated into the RNA-induced silencing complex [18, 19] in order to regulate gene expression by decreasing mRNA stability and/or block translation [20].

miRNAs have an enormous capacity of regulation and have been shown to play key regulatory roles in many biological processes like development, differentiation, homeostasis, carcinogenesis, apoptosis, regulation of immunity as well as in viral infections [21]. miRNAs have been identified in many species, from mammals to metazoans, funguses, plants and viruses. Studies on differential expression patterns of miRNAs have been carried out after virus infections, most of them in cell culture meanwhile a few of them have been performed in the natural host. In this proof-of-concept study, miRNA expression has been analyzed by using high throughput sequencing (HTS) in two tissues, spleen and submandibular lymph node (SLN), recovered at early times post-infection from pigs infected with two ASFV strains of different virulence and, on the other hand, the miRNA expression in these two tissues at different times post-infection in animals inoculated with the virulent strain E75 have been compared. The changes found in miRNA expression due to the virus infection could lead to a better understanding of the virus-host interactions and provide useful information to find new targets in order to control the infection. The information provided from this proof-of-concept study reinforces the application of this approach for future analysis using a higher number of animals and conditions.

## Methods

### Animals

Tissue samples analyzed in this study were derived from an experimental ASFV infection included in the investigation of ASFV pathogenesis and vaccine development [10]. Two different viruses were used. First, the España- 75 (E75) virulent strain (accession number FN557520), a very infectious and virulent strain causing almost a 100% mortality classified within p72 genotype I. This virus was isolate in an outbreak in Lerida in 1975 [1, 22]. And secondly, the E75CV1, an attenuated strain derived from the E75 strain obtained by cellular passage in a stable line of monkey kidney fibroblasts, the kidney of an adult male African green monkey (*Cercopithecus aethiops*) [23].

From the in vivo infection, available samples of four 8-week-old Landrace x Large White pigs were used. Submandibular lymph node (SLN) and spleen samples from two pigs intramuscularly inoculated with 10^4^ HAU of the E75 virulent strain [10] and SLN and spleen samples from two pigs inoculated with same amount (10^4^ HAU) of the E75CV1, were used. One animal inoculated with the virulent strain and two with the attenuated strain were necropsied at 3 days post-infection (dpi). At 7 dpi, one animal inoculated with the virulent strain was necropsied. All animal experiments conducted by Lacasta et al. (2015) were performed at CReSA facilities under biosafety level 3 (BSL-3) and all procedures were carried out in strict accordance with the guidelines of the institutional animal ethics committee of Universitat Autònoma de Barcelona (UAB), with the permit number DMAH-5796 for this specific study, according to the Spanish and European animal experimentation ethics law. Animals were euthanized following the European Directive 2010/63/EU, using an anaesthetic overdose of 60–100 mg of pentobarbital per kilogram of weight administered via the vena cava.

SLN and spleen samples were collected and immediately frozen at −80 °C.

### Homogenate and DNA- RNA extraction

Tissue samples of approximately 100 mg were homogenized with a pestle in 1 mL of Trizol (Thermo Fisher Scientific, Massachusetts, United States) to lyse the tissues and deactivate virus. Total RNA and DNA were isolated following the manufacturer’s protocol and resuspended in 25 and 200 μL of RNAse-, DNAse- and proteinase free water (Sigma- Aldrich, St. Louis, United States) for RNA and DNA, respectively.

### Real- time PCR

ASFV qPCR on both tissues was carried out in order to quantify viral load. SYBR Green qPCR was the selected procedure with pF1 primer (5′ CCTCGGCGAGCGCTTTATCAC 3′) as forward primer and pR2 (5′ GGAAACTCATTCACCAAATCCTT 3′) as the reverse one that were designed to target a highly conserved region within the p72 ORF as described by [24]. The mix for the qPCR contained 480 nM each primer, 12.5 μl Power SYBR® Green PCR Master Mix (Thermo Fisher Scientific) and 2.5 μl template. Nanopure autoclaved water was added to final volume of 25 μl. The conditions for the amplification were 10 min at 95 °C, 2 min at 50 °C and 40 cycles of 15 s at 95 °C and 1 min at 60 °C. Triplicates of each sample were used for the qPCR.

### RNA integrity and quantification

Total RNA was quantified using ND 1000 Nanodrop® Spectrophotometer (Thermo Fisher Scientific). RNA integrity and quality was analyzed using the 2100 Expert Bioanalizer (Agilent Technologies, Santa Clara, USA). Total and low size RNA measurements were carried out. Total RNA was calculated using the Eukaryote total RNA Nano Series II software and specific chips (Agilent Technologies, Santa Clara, USA), while low size RNA was measured using the Small RNA Nano software and specific chips (Agilent Technologies, Santa Clara, USA).

### Small RNA library construction

All procedures were carried out using the miRCat™ microRNA Cloning Kit (IDT, Iowa, USA) protocol as basis, with the modifications described in [25]. For the enrichment of the small RNA fraction, slices were cut from a 12% denaturing (7 M Urea) polyacrylamide gel, using a miSPIKE™ (IDT, Iowa, USA), as internal size marker. Small RNA fractions were purified using Performa DTR gel filtration cartridges (EdgeBio, Gaithersburg, USA). A double ligation was carried out using 3′ and 5′ linkers from miRCatTM kit (IDT, Coralville, USA) in two steps. The 3′ preadenylated linker was coupled to the small RNA fraction in a reaction in which the mix contained 1X Reaction Buffer for T4 Ligase (Thermo Fisher Scientific) without ATP, 0.1 mg BSA (Thermo Fisher Scientific), 1 U T4 RNA ligase (Thermo Fisher Scientific) and 2.5 μM 3′ linker. 6.5 μL small RNA fraction was added and incubated at 37 °C for 2 h. The 5′ linker was coupled in a mix with 1X ligation buffer (Thermo Fisher Scientific), 1 U T4 RNA ligase (Thermo Fisher Scientific) and 50 μM 5′ linker in presence of ATP. 7.5 μL 3′linked RNA was added to a final volume of 10 μL. The mixture was incubated for 2 h at 37 °C.

### RT- PCR

RT was carried out with Super Script III Reverse Transcriptase (Thermo Fisher Scientific). Initial pre incubation at 65 °C for 5 min with 0.5 mM of each dNTP, 0.5 μM Primer mir5 and 11 μL RNA were carried out. In a second step a mix was prepared with 1X First-Strand Buffer, 5 mM DTT, 0.2 U RNaseOUT and 10 U SuperScript III RT in a final volume of 20 μL, the mix was incubated at 55 °C for 90 min, and 70 °C for 15 min.

PCR amplifications were performed using Expand High Fidelity Plus PCR System (Roche Diagnostics, Basel, Switzerland). Briefly, 0.05 U Expand High Fidelity Plus Enzyme Blend, 1X Expand High Fidelity Plus Reaction Buffer with 1.5 mM MgCl_2_, 0.2 μM Primers A5 and B3 (included multiplex identifiers at the 5′ end), 0.2 μM of each dNTP, 4 μL cDNA and RNAse free water to a final volume of 50 μL. PCR conditions were 94 °C for 3 min, 20–25 cycles at 94 °C for 30 s, 57 °C for 45 s and 72 °C for 1 min followed by 71 °C for 7 min. The number of cycles was optimized for each library to avoid saturation. The resulting PCR was purified with the QIAquick PCR purification kit (Quiagen, Hilden, Germany).

Cloning in the pGEM-T Easy Vector System II Kit (Promega Corporation, Wisconsin, United States) and posterior Sanger sequencing with the ABI-PRISM 3700 (Thermo Fisher Scientific) were carried out to check the libraries construction prior to the HTS.

### High throughput sequencing

PCR amplifications from each library were quantified using Qubit™ fluorometer, Quant-IT™ (Invitrogen™, Carlsbad, USA) and prepared to a 10^11^ DNA molecules/μL. As they included multiplex identifiers, they were equimolecular pooled to continue in one reaction. Ion Torrent adapters were ligated to 30 ng of pooled DNA and libraries were then amplified with Ion Torrent primers for 8 cycles and size selected (2% E-Gel Size Select, Invitrogen). Libraries were sequenced following the manufacturer’s protocol with Ion PGM™ sequencer (Thermo Fisher Scientific, Massachussetts, USA) at DNA sequencing facilities at CRAG (Bellaterra, Spain). Software version for base calling was Torrent-Suite v2.0.1, (Thermo Fisher Scientific, Massachussetts USA). Sequencing data were deposited at European Nucleotide Archive (ENA, http://www.ebi.ac.uk/ena/) [26] with the accession number PRJEB17083.

### Sequence processing scheme and miRNA expression statistics

Primer sequences were trimmed and only those insert sequences between 15 and 29 nucleotides and with total number of sequences ≥3 were kept for further analysis.

For porcine miRNA profiling, sequences were compared to all available miRNA sequences (miRBase v21) using local Blast. Parameters were set to 100% identity and up to 4 mismatches allowed at the end of the sequences to solve the miRNA variability on 3′ and 5′ ends [27].

Differences in host miRNA expression were assessed. The total number of sequences obtained for each porcine miRNA was normalized by library size (in counts per thousand) and, then, averaged by group. Fold changes (FC) between groups were calculated using normalized data. Only miRNAs up or down-regulated for all individuals per group were taking into account. miRNAs were considered differentially expressed (DE) with a FC > 5, and highly expressed with a copy number > 80, according with a previous study from our group [28].

### miRNA target prediction and biological functions

For the in silico study of the potential targets, the DIANA-microT-CDS v5.0 web server with an adjusted threshold of 0.7 was used [29, 30]. There were no porcine genes in the databases, so the study was done using the human genome assuming sequence conservation [31]. In order to identify potential targets in the viral genome, the miRanda program was used with the following parameters: -sc 140 –en 20. Potential targets were then studied employing Web-based Gene Set Analysis Toolkit (WebGestal) [32, 33]. The analysis was done using the Kyoto Encyclopedia of Genes and Genomes Database (KEGG) to check the biological pathways in which miRNAs are involved. The selected parameters for the study were the multiple test adjustment by Benjamini and Hochberg [34] and the significance level set at 0.05.

Finally, Cytoscape 3.2.1 software [35] was used in order to build the gene regulatory networks formed by the DE miRNAs and their cellular and viral target genes.

## Results

### Animal infection

Pigs inoculated with the E75CV1 attenuated strain developed no clinical signs while pigs inoculated with the E75 virulent strain developed typical African swine fever clinical signs [10]. Samples from three animals necropsied at 3 dpi as early times of infection and from one pig inoculated with the virulent strain euthanized at 7 dpi because of the development of severe clinical signs were analyzed. These time points reflect early times of infection (3dpi) where low viral load and mild clinical/ histopathological signs were observed, and, on the other hand late times of infection (7 dpi) where very high viral load was detected with severe and lethal clinical and histopathological signs. At 3 dpi animals presented no fever but a reduction of their activity and mild awkwardness in their movement with few hemorrhages or cyanotic areas and low dyspnea. Histopathology showed mild bleeding, splenic infarcts, hyperplasia, necrosis, lymphocytolysis or vascular damage in spleen. At 7 dpi animals presented high fever with mild movement or even immobile, high percentage of cyanotic areas, cutaneous necrosis and hemorrhages, bloody faeces and dyspnea. Histopathology showed bleeding, lymphoid depletion, splenic infarcts, necrosis and massive hemorrhages in spleen). Ct values in tissue samples determined by qPCR are indicated in Table 1. DNA from attenuated virus was not detected at 3 dpi by qPCR. Animals included in the study of Lacasta et al. (2015), inoculated with the attenuated strain and maintained until 31 dpi showing neither viremia nor clinical signs of acute ASF except fever episodes at 7 dpi that reached undetectable levels by 28 dpi.

**Table 1:** Ct values from ASFV inoculated pigs

	Animal number			
Tissue	1	2	3	4
SLN	31.99	27.81	–	–
Spleen	27.71	24.6	–	–

Animals 1 and 2 were inoculated with the E75 virulent strain, animals 3 and 4 were inoculated with the E75CV1 attenuated strain

### miRNA sequence annotation

RNAs from SLN and spleen were used to prepare small RNA libraries from animals euthanized at 3 and 7 dpi. A total of 8 small RNA libraries from both tissues were constructed and sequenced with an Ion Torrent PGM sequencer. From the 301,802 total reads obtained, after trimming the adaptor sequences, the number of inserts comprising 15 to 29 nt were 105,156. Sequences were aligned to miRBase database (v.21) and a total of 66,599 hits were obtained, corresponding to 366 entries in miRBase.

### Analysis of the miRNA expression in animals infected with the virulent ASFV strain at different times post-infection

The normalized count of sequencing reads (reads/total sequencing tags in the library) could be used to quantify miRNA expression levels among early (3 dpi) and late (7 dpi) in both analyzed tissues. miRNAs were considered differentially expressed (DE) with a FC > 5, and highly expressed with a copy number > 80, according with a previous study from our group [28].

From the 14 miRNAs expressed at higher levels (>80 reads) in spleen, 9 miRNAs (64.3%) were DE, two were up-regulated at 7 dpi when compared with 3 dpi (miR-451 and miR-145-5p) and 6 down-regulated (Table 2). Interestingly, miR-451 and miR-145-5p were the most represented miRNAs among all miRNAs, with more than two and one thousand counts found at day 7 for E75-infected pigs, respectively. Among the down regulated miRNAs, miR-126-5p presented the highest FC differences with 225 FC. In SLN, from the 25 miRNAs more expressed (>80), six miRNAs (24%) were DE, two up-regulated at 7 dpi when compared with 3 dpi (miR-181a and miR-145-5p) and four down-regulated (Table 3). As has been shown in spleen, miR-126-5p was the miRNA with the highest FC (41.84) in SLN. A more conserved miRNA expression pattern was observed in SLN than in spleen.

**Table 2:** Differences in the expression of miRNAs in spleen between virulent ASFV infected animals

miRNA	7 dpi/3 dpi	Reads
bta-miR-451	46.15	2305
ssc-miR-145-5p	12.39	1212
ssc-miR-92a	−21.02	445
ssc-miR-23a	−7.92	285
ssc-miR-92b-3p	−13.79	271
hsa-miR-25-3p	−2.10	203
ssc-miR-126-5p	−224.86	202
ssc-miR-23b	−16.74	152
ssc-miR-26a	−1.61	151
ssc-miR-122	106/0	106
ssc-miR-125b	−3.29	96
ssc-miR-21	1.83	92
ssc-miR-181a	1.06	88
bfl-miR-92c	−13.87	86

The most represented miRNAs (CN >80) are indicated. miRNAs were considered DE when FC was higher than 5 between 7 dpi and 3 dpi samples of infected pigs

**Table 3:** Differences in the expression of miRNAs in SLN between virulent ASVF infected animals

miRNA	7dpi/3 dpi	Reads
ssc-miR-126-3p	−9.32	1892
ssc-miR-23a	−2.49	1576
ssc-miR-126-5p	−41.84	622
ssc-miR-23b	−5.14	586
ssc-miR-26a	−1.06	530
ssc-miR-125b	1.54	447
ssc-miR-92a	−1.05	440
ssc-miR-21	4.69	431
hsa-let-7b-5p	2.67	423
hsa-miR-25-3p	−1.05	412
ssc-miR-92b-3p	2.30	312
ssc-miR-15b	1.32	284
ssc-miR-150	1.29	257
ssc-miR-99a	−1.54	199
ssc-miR-10b	−2.07	180
ssc-miR-30e-5p	−2.80	174
ssc-miR-30d	−7.11	161
ssc-miR-100	−1.29	137
ssc-miR-181a	5.36	131
ssc-miR-16	4.20	116
ssc-miR-99b	−2.97	115
ssc-miR-378	−2.92	113
bfl-miR-92c	1.00	109
ssc-miR-145-5p	5.87	95
hsa-miR-500a-5p	−4.09	94

The most represented miRNAs (CN >80) are indicated. miRNAs were considered DE when FC was higher than 5 between 7 dpi and 3 dpi in samples of infected pigs

### Analysis of miRNA expression in attenuated and virulent ASFV strains infected animals at early times post- infection

The normalized count of sequencing reads (reads/total sequencing tags in the library) could be used to quantify miRNA expression levels among virulent and attenuated ASFV infected pigs at early times post-infection (3 dpi). miRNAs were considered DE with a FC > 5 and when the up- or down-regulation was conserved for both animals infected with the E75CV1 strain.

From the 22 more expressed miRNAs (>80 reads) in spleen, 7 miRNAs (31.8%) were DE, four up-regulated in virulent ASFV infected animal (miR-92a, miR-126-5p, miR-92c and miR-30e-5p) and 3 down-regulated (miR-125b, miR-451 and miR-125a) (Table 4). In SLN, from the 33 more expressed miRNAs (>80), three miRNAs (9.1%) were DE, two up-regulated in virulent infected animal (miR-30e-5p and miR-500a-5p) and one down-regulated (miR-125a) (Table 5). miR-303-5p and miR-125a presented the same regulation in both tissues analyzed, up and down regulated, respectively. In this case, the miRNA expression seems also to be more conserved in SLN than in spleen at that time post-infection.

**Table 4:** Differences in the expression of spleen miRNAs between virulent and attenuated ASFV infected animals

miRNA	Virulent/ Attenuated	Reads
ssc-miR-125b	−6.64	1496
ssc-miR-23a	−1.42	1266
ssc-miR-23b	−1.31	642
ssc-miR-92a	13.06	508
ssc-miR-92b-3p	3.29	478
bta-miR-451	−5.80	446
ssc-miR-125a	−7.59	440
ssc-miR-145-5p	−2.63	418
ssc-miR-126-5p	12.42	250
hsa-miR-25-3p	3.14	213
ssc-miR-99a	−2.05	211
ssc-miR-378	−2.14	194
hsa-let-7b-5p	1.06	172
ssc-miR-126-3p	2.03	162
ssc-miR-100	−3.03	145
ssc-miR-30d	2.04	130
ssc-miR-26a	2.86	129
ssc-miR-99b	−1.85	127
ssc-miR-21	−1.59	93
bfl-miR-92c	13.58	90
ssc-miR-30e-5p	5.70	90
ssc-miR-150	−1.02	84

The most represented miRNAs (CN >80) are indicated. DE miRNA were considered as those suffering >5 FC

**Table 5:** Differences in the expression of SLN miRNAs between virulent and attenuated ASFV infected animals

miRNA	Virulent/ Attenuated	Reads
ssc-miR-126-3p	1.55	2601
ssc-miR-23a	1.17	2424
ssc-miR-125b	−2.55	1030
ssc-miR-92b-3p	−4.41	869
ssc-miR-23b	1.39	853
ssc-miR-126-5p	1.89	847
ssc-miR-92a	−1.10	646
ssc-miR-26a	2.49	614
hsa-let-7b-5p	−1.21	568
hsa-miR-25-3p	1.68	509
ssc-miR-21	1.03	452
ssc-miR-150	1.06	355
ssc-miR-15b	1.66	333
ssc-miR-99a	−1.34	332
ssc-miR-125a	−6.91	291
ssc-miR-10b	1.17	261
ssc-miR-100	−1.42	238
ssc-miR-30d	1.79	223
ssc-miR-30e-5p	6.83	183
ssc-miR-99b	1.02	182
ssc-miR-191	−2.32	164
bfl-miR-92c	1.19	145
ssc-miR-145-5p	−2.48	140
ssc-miR-378	2.51	133
ssc-miR-181a	1.05	131
ssc-miR-16	1.16	115
ssc-miR-204	1.09	115
ssc-miR-218	1.28	103
hsa-miR-500a-5p	5.74	101
hsa-miR-29c-5p	−1.09	96
ssc-miR-374a-5p	2.09	89
ssc-miR-186	2.57	84
efu-miR-126	−1.34	82

The most represented miRNAs (CN >80) are indicated. DE miRNA were considered as those suffering >5 FC

Interestingly, only two miRNAs were equally regulated in spleen and SLN when comparing tissues from virulent and attenuated ASFV infected animals at 3 dpi. Thus, while the ssc-miR-125a showed a down-regulation in its expression, ssc-miR-30e-5p was overexpressed in tissues infected with virulent ASFV when compared to animals infected with the attenuated strain.

### miRNA target prediction and biological pathways analysis

Target predictions were carried out for DE miRNAs. Ten miRNAs were selected for target prediction according to the highest representation by tissue and conditions: miR-23a, miR-30e-5p, miR-92a, miR-122, miR-125b, miR-126-5p, miR-145-5p, miR-125a, miR-451 and miR-126-3p.

From these ten miRNAs a total of 8774 target genes were identified (Additional file 1). Cellular target genes were functionally analyzed through the KEGG pathways database (Table 6). For 7 miRNAs: miR-23a, miR-30e-5p, miR-92a, miR-122, miR-125b, miR-126-5p and miR-125a, significant pathways were found related to immune response such as B and T cell receptor signaling pathway, natural killer cell mediated cytotoxicity or Fc gamma R-mediated phagocytosis and with some processes related to the pathogenesis and virus-host interaction, like desencapsidation, apoptosis inhibition, autophagy or host DNA damage response. For miR-451, miR-126-3p and miR-145-5p, no pathways were identified, although some of the target genes have been related to ASFV infection.

**Table 6:** Genome pathways predicted for selected miRNAs from Kyoto Encyclopedia of Genes and Genomes

miRNA	Pathways
miR-23a	Endocytosis
	Regulation of actin cytoskeleton
	RIG-I-like receptor signaling pathway
	Leukocyte transendothelial migration
	Fc gamma R-mediated phagocytosis
	Protein processing in endoplasmic reticulum
	NOD-like receptor signaling pathway
	Cell adhesion molecules (CAMs)
	Chemokine signaling pathway
	Apoptosis
	T cell receptor signaling pathway
	Hematopoietic cell lineage
	Phagosome
	Fc epsilon RI signaling pathway
miR-30e-5p	Regulation of actin cytoskeleton
	Natural killer cell mediated cytotoxicity
	T cell receptor signaling pathway
	B cell receptor signaling pathway
	Focal adhesion
	Protein processing in endoplasmic reticulum
	Endocytosis
	Fc epsilon RI signaling pathway
	Apoptosis
	Regulation of autophagy
	Fc gamma R-mediated phagocytosis
	Leukocyte transendothelial migration
	Cell adhesion molecules (CAMs)^a^
	Chemokine signaling pathway
	mTOR signaling pathway^a^
	Toll-like receptor signaling pathway
	RIG-I-like receptor signaling pathway
miR-92a	Focal adhesion
	Regulation of actin cytoskeleton
	Endocytosis
	Fc gamma R-mediated phagocytosis
	Cell adhesion molecules (CAMs)^a^
	Leukocyte transendothelial migration
	Chemokine signaling pathway
	B cell receptor signaling pathway
	T cell receptor signaling pathway
miR-122	Antigen processing and presentation
	Endocytosis
	T cell receptor signaling pathway
miR-125b	Toll-like receptor signaling pathway
	Endocytosis
	Apoptosis
	Chemokine signaling pathway
	Regulation of actin cytoskeleton
	Fc epsilon RI signaling pathway
	NOD-like receptor signaling pathway
	Cell adhesion molecules (CAMs)^a^
	T cell receptor signaling pathway
	Natural killer cell mediated cytotoxicity
	Hematopoietic cell lineage
miR-126-5p	Focal adhesion
	Regulation of actin cytoskeleton
	T cell receptor signaling pathway
	Protein processing in endoplasmic reticulum
	Natural killer cell mediated cytotoxicity
	B cell receptor signaling pathway
	Fc epsilon RI signaling pathway
	Chemokine signaling pathway
	Apoptosis
	Endocytosis
	Regulation of autophagy
miR-145-5p	Regulation of autophagy
	Protein processing in endoplasmic reticulum
miR-125a	Toll-like receptor signaling pathway
	Endocytosis
	Apoptosis
	Chemokine signaling pathway
	Antigen processing and presentation
	Regulation of actin cytoskeleton
	Fc epsilon RI signaling pathway
	NOD-like receptor signaling pathway
	Hematopoietic cell lineage
miR-451	–
miR-126-3p	–

All target genes reflected in this table are related to the immune system and ASFV pathogenesis. ^a^Signal transduction

### Gene regulatory network

**Fig. 1:**
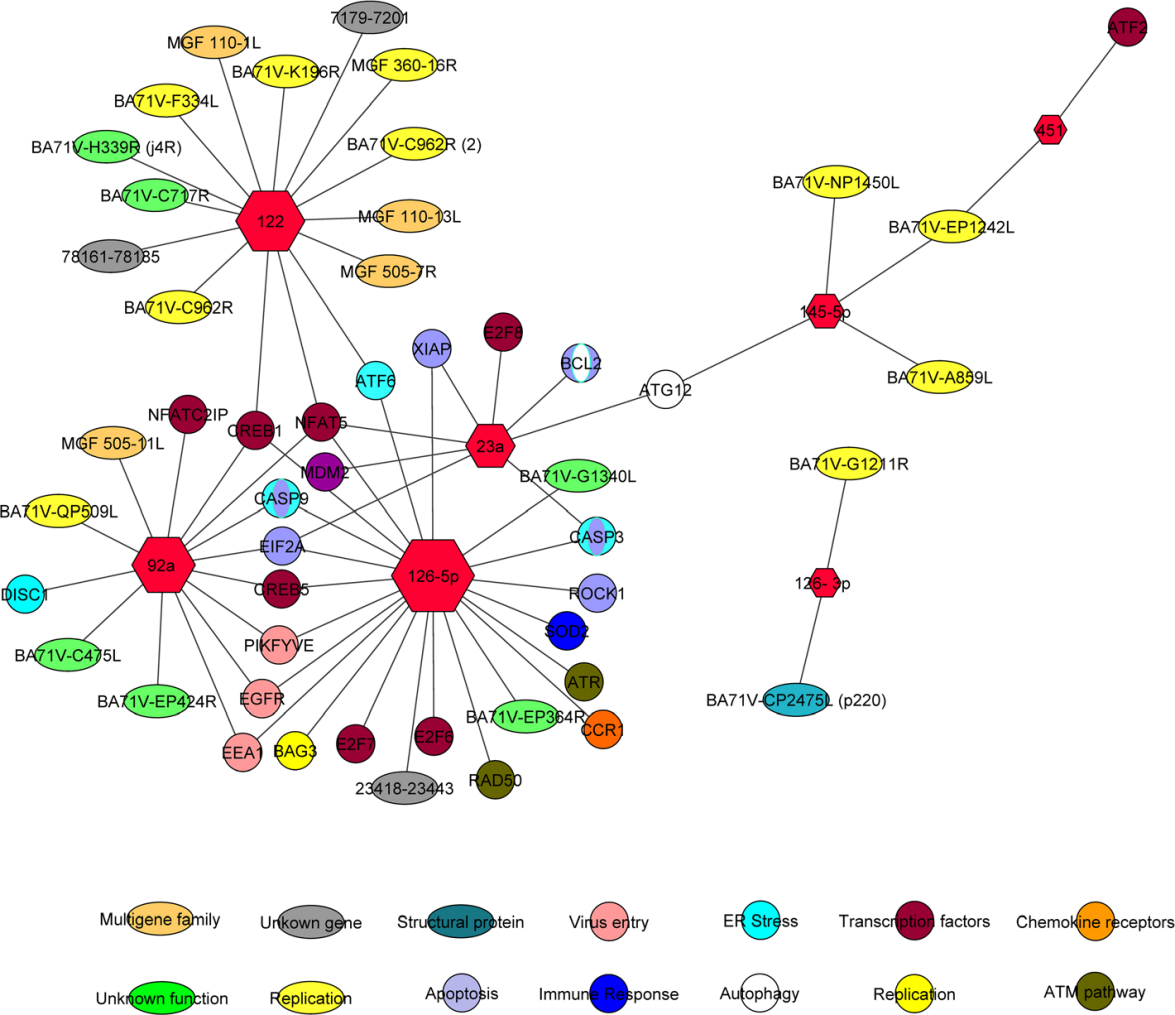
Network of DE miRNA interactions in animals infected with the virulent strain at different time points. ASFV genes and porcine genes involved in virus- host interactions are represented. Hexagon size is proportional to the miRNAs number of interactions. Circles indicate cellular genes and ovals indicate viral genes. Colors indicate the main/s pathway/s in which they are involved or the described function of the viral genes

**Fig. 2:**
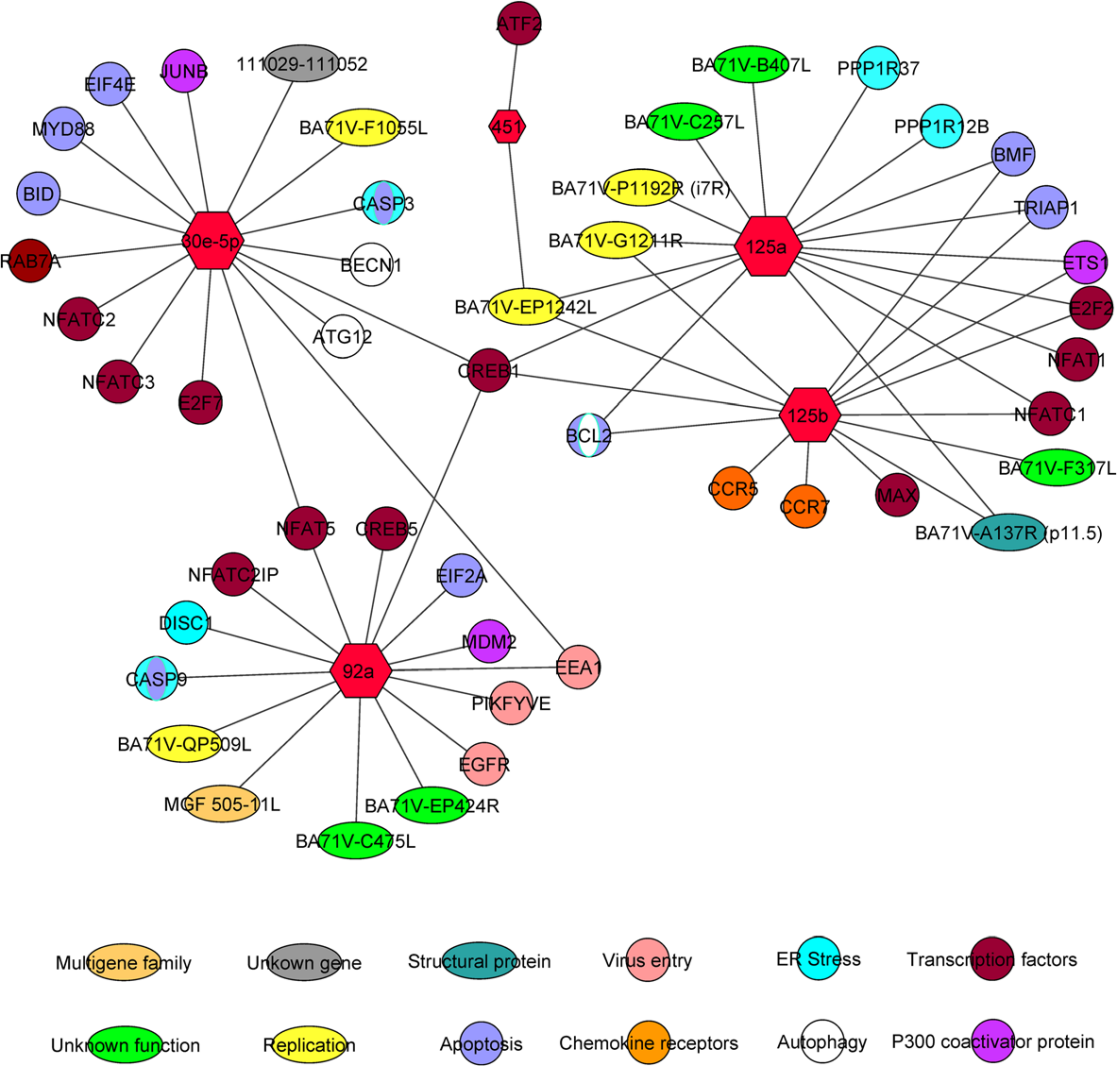
Network of DE miRNAs interactions in animals infected with virulent strain and animals infected with attenuated strain. ASFV genes and porcine genes at 3 dpi involved in virus- host interactions are represented. Hexagon size is proportional to the miRNAs number of interactions. Circles indicate cellular genes and ovals indicate viral genes. Colors indicate the main pathway/s in which they are involved or the described function of the viral genes

**Fig. 3:**
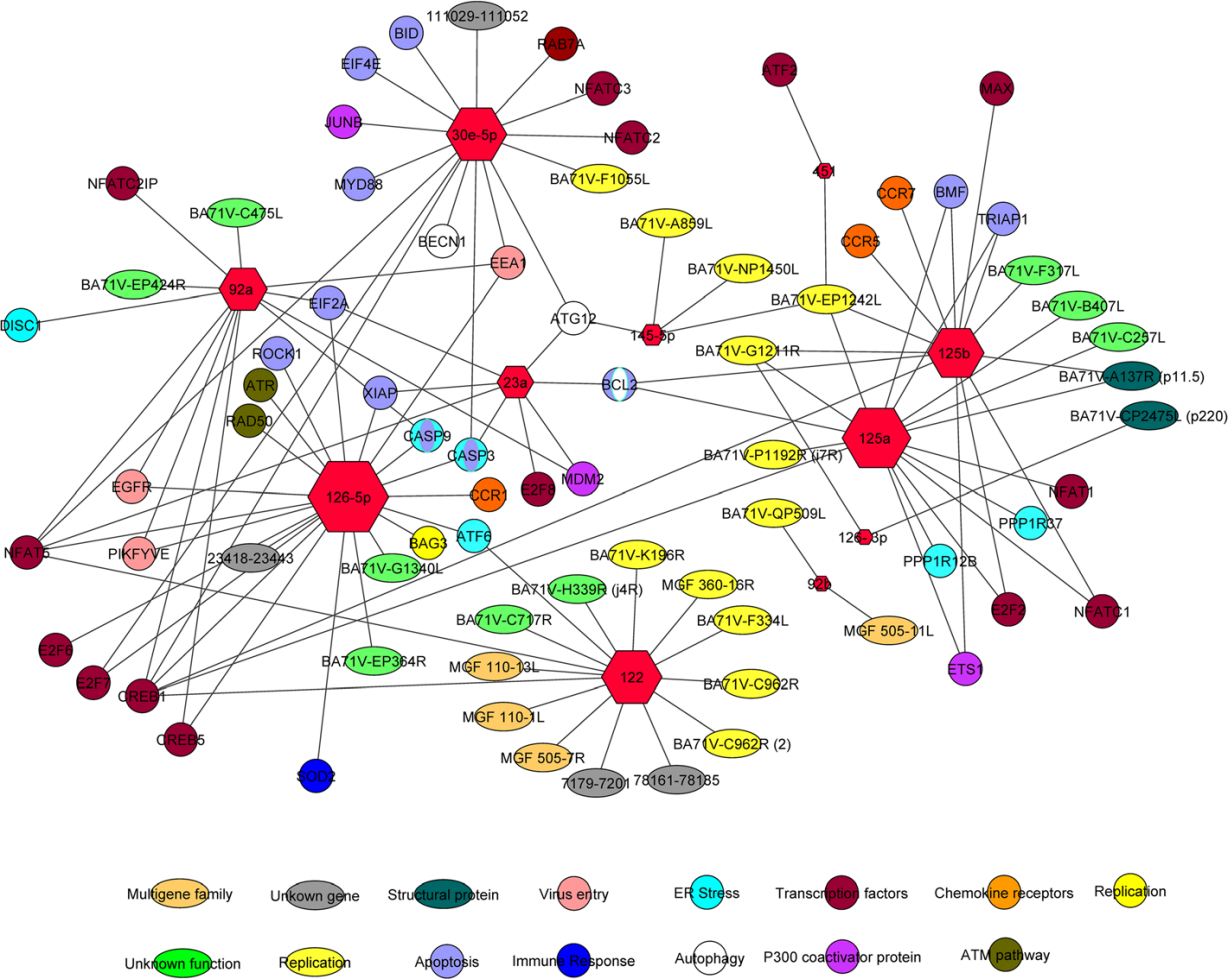
Global miRNA network interaction. Related DE miRNAs in both approaches with cellular target genes involved in ASFV infection and with viral genes. Hexagon size is proportional to the miRNAs number of interactions. Circles indicate cellular genes and ovals indicate viral genes. Colors indicate the main pathway/s in which they are involved or the described function of the viral genes

DE miRNAs were analyzed with the miRanda algorithm in order to identify the possible targets in the ASFV genome. For miRNA expression in animals infected with the virulent ASFV at different dpi, a total of 25 interactions were found for the seven DE miRNAs and the annotated 165 genes in ASFV genome (Fig. 1). Also, 42 interactions were identified with the porcine genes involved in virus-host interactions such as: apoptosis, chemokine receptors, endoplasmic reticulum (ER) stress, transcription factors, autophagy, ATM pathway, virus entry, immune response and replication. For miRNA expression in attenuated and virulent infected animals at early times post-infection, 16 interactions were found for the five DE miRNAs and the annotated genes in ASFV genome (Fig. 2) and 45 interactions were identified with the porcine genes involved in virus-host interaction, like: chemokine receptors, ER stress, apoptosis, transcription factors, autophagy, virus entry and P300 coactivator protein. In a global approach, considering all DE miRNAs by ASFV infection condition, 37 interactions were found between 12 DE miRNAs included and the annotated 165 genes in ASFV genome (Fig. 3). Also, 76 interactions were identified with the porcine genes involved in virus-host interaction as previously described for both conditions.

## Discussion

This study has been carried out in order to decipher the presence and the potential role of the miRNAs during in vivo ASFV infections and virus pathogenesis. The miRNA DE pattern has been studied for many viruses affecting pigs, like TGEV, PPV, Porcine cytomegalovirus, ADV, PCV2 and PRRSV [25, 36–40], principally by using cell culture. Only a few studies have been carried out analyzing the expression pattern in the natural host. This in vivo expression pattern analysis could be considered a more reliable method to replicate the natural conditions in which infection and host-pathogen interactions take place [25, 28, 41]. This proof-of-concept study has allowed the analysis of differences in miRNA expression at different times post-infection with a virulent strain and the expression pattern induced by virulent and attenuated strains at an early time post-infection. However, further functional analysis is needed to support and confirm these predictions. On the other hand, the use of a high number of animals could provide a more robust results. Nevertheless, we consider that the selected samples are representative of each group, taking into account that the well characterized virological, clinical and immunological parameters of each group were similar [10].

As previously described [10], animals inoculated with virulent strain E75 developed clinical signs compatible with acute ASF detectable from 4 dpi and reaching its peak at 7 dpi, while animals inoculated with the attenuated strain E75CV1 developed no clinical signs and no viral genome was detected at any time post-infection in the selected tissues.

When comparing different times post-infection with the E75-virulent strain, more than half of the spleen miRNAs expressed at higher levels (highest copy number), were DE (64.3%), when considering as DE those miRNAs with a FC > 5. Interestingly, two were up-regulated at 7 dpi (miR-451 and miR-145-5p), while the rest were down-regulated. In SLN, a minor proportion (24%) of the miRNAs were DE. This matches with the differential transcription and protein profile observed in gastrohepatical lymph node at these two times post-infection [10].

Regarding the differential expression pattern between the virulent E75 and the attenuated E75CV1 at early times post-infection (3 dpi), again, a higher differential expression has been detected in spleen while a more conserved pattern was observed in SLN. In spleen, 31.8% (7 out of 22) of the most represented miRNAs (CN > 80) were DE. In SLN, the percentage decreased to 9.1% (3 out of 33). Although both spleen and lymph nodes are targets for virus replication, the different vascularization, anatomical organization and function might explain this differential regulation.

In SLN, the four DE miRNAs most represented at different times after the infection with the ASFV virulent strain, ssc-miR-126-3p, ssc-miR-126-5p, ssc-miR-23b and ssc-miR-30d were down-regulated at 7 dpi, coinciding with the massive detection of cytokines and other immune mediators, with a marked leucopenia, hemorrhages and with the death of the infected animals [10]. The cytokine storm classically described during the acute phase of ASF might be explained at least partially by the marked down-regulation of these specific miRNAs, previously described as key regulators of genes involved in the regulation of the immune response [42–44] including: hematopoietic cell lineage, complement and coagulation cascades, platelet activation, Toll-like receptor signaling pathway, NOD-like receptor signaling pathway, RIG-I-like receptor signaling pathway, cytosolic DNA-sensing pathway, natural killer cell mediated cytotoxicity, antigen processing and presentation, T cell receptor signaling pathway, B cell receptor signaling pathway, Fc epsilon RI signaling pathway, Fc gamma R-mediated phagocytosis, leukocyte transendothelial migration, intestinal immune network for IgA production and chemokine signaling pathway.

miR-451 was the most represented miRNA in spleen of the virulent infected animals and is also DE between 3 and 7 dpi. Thus, miR-451 is down-regulated at 3 dpi and up-regulated at 7 dpi. Interestingly, miR-451, was also DE at 3 dpi in spleen of E75CV1-infected pigs, on this occasion, showing a clear up-regulation. A similar expression profile has been described for some key mediators of the innate immune response [10], albeit the target prediction analysis showed that this miRNA was potentially able to interact with 37 genes, no significant pathways related to immune response or pathways involved in ASFV-host interaction have been identified.

miR-145-5p was up-regulated at 7 dpi in the spleen and SLN of the animal infected with the virulent strain. As described for miR-451, no pathways related to immune response have been predicted for this miRNA, among the 293 target genes identified. Nevertheless, one pathway related to ASFV-cell interaction, regulation of autophagy through ATG12, has been found. The overexpression of miR-145-5p at the time of maximal virus production (7 dpi) might contribute to an inhibition of autophagy to facilitate ASFV replication and to avoid virus clearance, as has been postulated before [45].

miR-92a was DE at different times in the spleens of animals infected with the virulent strain E75, with its expression being up-regulated at 3 dpi. When comparing the spleens of animals infected with the virulent or attenuated strains at 3 dpi, there was also DE, where the expression is up-regulated by the virulent strain. Target prediction showed 1192 regulated genes and the pathway analysis revealed that it was involved in five pathways related to immune response, B and T cell receptor signaling pathways, Fc gamma R-mediated phagocytosis, chemokine signaling pathway and leukocyte transendothelial migration. Other associated pathways are endocytosis and regulation of actin cytoskeleton. The participation of actin in the endocytosis process has been proposed as stimulator for the entry of large viruses, like vesicular stomatitis virus [46] and also for ASFV [47], therefore, we anticipate that miR-92a could be involved in the entry process of ASFV, corresponding with the peak of virus replication at 7 dpi.

Another miRNA showing a different expression pattern in spleen between 3 dpi and 7 dpi was miR-23a. This miRNA was down-regulated at 7 dpi. A large number of significant pathways were found associated with miR-23a target genes, some of them related to the immune response, such as RIG-I-like receptor signaling pathway leukocyte transendothelial migration, Fc gamma R-mediated phagocytosis, NOD-like receptor signaling pathway, chemokine signaling pathway, T cell receptor signaling pathway, hematopoietic cell lineage and Fc epsilon RI signaling pathway. On the other hand, pathways related to the entry of the virus, like endocytosis and regulation of actin cytoskeleton, as well as other related to the apoptotic machinery were found. Among the target genes predicted, Bcl2 and Caspase 3 were two of the most significant genes involved in apoptosis [48]. Also, this miRNA regulates eIF2a which is involved in ER stress processes [49]. Thereby, miR-23a can play a role in the apoptosis induced by ASFV and in the immune response against the virus. Compared with 3 dpi, miR-126-3p was down-regulated in SLN at 7 d after infection with the virulent E75 strain. Only 20 genes were identified as possibly regulated by miR-126-3p, and no related immunological pathways were detected.

miR-126-5p was down-regulated both in SLN and spleen at 7 dpi after infection with the virulent E75 strain, compared with 3 dpi, and could regulate genes related to the immune response and many aspects of the virus-host interaction like entry, apoptosis, regulation of autophagy, ER stress or chemokine receptors. This regulation could be in agreement with our observation about miR-126-5p down-regulation that paralleled the cytokine storm found during the last stages of ASF acute disease.

miR-125b was DE in spleen at 3 dpi between virulent and attenuated strains, being down-regulated by the virulent E75 strain. miR-125b was shown to interact with a large number of genes, thus, some pathways related to immune response have been identified for this miRNA. In addition, it has also been associated with pathways involved in the ASFV-cell interaction such as endocytosis, regulation of actin cytoskeleton and apoptosis [45]. Target prediction analysis showed that miR-125b can interact with Bcl2. Down-regulation of Bcl2 by miR-125b might contribute to the inhibition of the early apoptosis of E75 infected macrophages thus favoring the success of its replication.

It is worth noting the differential regulation of miR-30e-5p and miR-125a, two miRNAs that follow opposite expression profiles that interestingly were conserved for both tissues studied. Thus, the expression of miR-30e-5p was up-regulated in SLN and spleen at 3 dpi infected with the virulent E75 strain, compared to the attenuated E75CV1, while miR-125a was down-regulated, perhaps playing opposite regulatory roles during the fine equilibrium that separate attenuation from virulence. The fact that all functions associated with miR-125a are shared by miR-30e-5p, allowed us to hypothesize about the relevant roles for pathways specifically regulated by overexpressing miR-30e-5p early after infection with virulent E75 ASFV strain, and contributing to the inhibition of key macrophage innate defensive mechanisms such as mTOR, toll-like receptors, RIG-I-like receptors, cell adhesion molecules, chemokine signaling pathways, Fc gamma R-mediated phagocytosis, leukocyte transendothelial migration or CAMs. Down-regulation of any of these pathways might contribute to successful evasion of the innate immune response.

miR-122 was not expressed in spleen at 3 dpi of animal infected with the virulent E75. Conversely, its expression was notably high at day 7pi. A small number of pathways related with immune response have been observed for this miRNA and it has only been associated with the endocytosis pathway.

Thus, from this analysis we can conclude that ASFV modifies miRNA expression patterns involved in the immune response and might contribute to the course of the disease during the infection.

From the gene network analysis, we found that some miRNAs like miR-451 and miR-145-5p, which are the most represented DE miRNAs in spleen, and are highly up-regulated at 7 dpi, are associated with the viral gene 1242 L. This gene, also regulated by miR-125a and miR-125b, is involved in RNA transcription and processing [50]. ASFV genome transcription is carried out without using the host RNA polymerase II. The described gene function for this gene is RNA polymerase subunit 2 making it a candidate to explore as a target to regulate viral replication.

miR-125a additionally targets the viral gene P1192R, which codes for a type II DNA topoisomerase that is essential for viral replication and/or transcriptional events [51]. In addition, this topoisomerase has been proposed as a potential target to control the disease by using poisons and inhibitors against this enzyme [52]. Down-regulation of miR-125a might contribute to evade blockade of P1192R, thus contributing to the efficient replication of the E75 virulent virus.

Interestingly, the three miRNAs that interact with Bcl2 (miR-23a, miR-125a and miR-125b), do not theoretically regulate A179L, the homologous viral gene of the apoptosis inhibitor gene Bcl2 [53]. In spite of the high degree of structural similarity to all Bcl-2 proteins [54], the sequence divergence between both genes might explain this differential regulation and might contribute to the successful apoptosis inhibition of the infected macrophage until the virus cycle is finished.

miR-92a interacts with the three genes identified as being involved in ASFV entry, EEA1, EGFR and PIKFYVE [55, 56] both in the virulent infection at 3 and 7 dpi and between attenuated and virulent at 3 dpi. Interestingly, this miRNA was found in much higher levels (thirteen times) in spleen of the animal inoculated with the virulent strain at 3 dpi compared to spleens of animals inoculated with the attenuated strain. This difference of expression could be involved in the differences in the dynamics of virus infection depending on its virulence and, is in accordance with the recent study where the diminution of PIKFYVE decreased the ASFV infectivity and viral production [57].

Target prediction for miR-122 revealed that it interacts with 12 different viral genes classified in multigene family, replication, genes with unknown function and unknown genes. This miRNA interacts with MGF 360-16R and it has recently been described that this viral multigene family component modulates host innate responses by determining the tropism, virulence and suppression of type I IFN response [57]. In addition, miR-122, together with miR-126-5p, target ATG6, which is activated by ASFV. The virus uses the ER as a site of replication and this process of activation of ATF6 can trigger ER stress and the unfolded protein response (UPR) of the host cell [58]. miR-122 is the DE miRNA with the highest number of viral target genes and none of these is regulated by other miRNAs. On the other hand, miR-122 is not express in spleen from an infected animal with the virulent E75 strain at 3 dpi, while at 7 dpi its expression was notably increased. In addition, it is well known that miR-122 plays a key role in Hepatitis C virus infection [59]. Accordingly, this miRNA could be involved in the regulation of the “success of the ASFV infection”.

To our knowledge, this is the first time that a deep sequencing approach has been used to study miRNA gene expression in pigs infected with ASFV. However, a larger number of animals as well as functional analysis would be necessary to support the results obtained in this study. These investigations could confer a more accurate vision of the relation between miRNAs with porcine target genes and ASFV genes to help decipher the role of miRNAs in ASFV infection.

## Conclusion

In this proof-of-concept study, we have been able to identify differentially expressed porcine miRNAs during ASFV infection. These DE miRNAs have been observed at different times post ASFV infection and between different strains with differences in virulence. From these miRNAs, their host and viral target genes and the pathways related to the disease in which they are involved have been analyzed. Therefore, this information will contribute to our knowledge on ASFV infections and help clarify host- pathogen interactions, the mechanisms of the viral infection and the development of the disease.

## Additional file

Additional file 1: Target genes identified from DE miRNAs. (DOCX 43 kb)

## Abbreviations

ASF: African swine fever
ASFV: African swine fever virus
CAMs: Cell adhesion molecules
CN: Copy number
DE: Differentially expressed
DPI: Days post-infection
ENA: European nucleotide database
ER: Endoplasmic reticulum
FC: Fold change
HTS: High throughput sequencing
KEGG: Kyoto encyclopedia of genes and genomes
miRNA: MicroRNA
OIE: World Organization for Animal Health
Pre-miRNA: Precursor miRNA
Pri-miRNA: Primary miRNA
SLN: Submandibular lymph node
UPR: Unfolded protein response
WebGestalt: WEB-based GEne SeT AnaLysis Toolkit
