# Supplementary material for: Differential expression of porcine microRNAs in African swine fever virus infected pigs: a proof-of-concept study
Source: Virol J. 2017 Oct 17;14:198. doi: 10.1186/s12985-017-0864-8 (PMC5646143; doi:10.1186/s12985-017-0864-8)
Supplement: Additional file 1: — Target genes identified from DE miRNAs. (DOCX 43 kb) [file 12985_2017_864_MOESM1_ESM.docx]

| **miRNA** | **Predicted target genes** |
| --- | --- |
| miR-30e-5p | CELSR3, BRWD3, TBC1D10B, C9orf72, AC130352.1, PCDH10, GLCCI1, CPNE8, KIAA1549, RFX6, IP6K3, EED, SEMA3A, SCN2A, LHX8, COL25A1, PFN2, MMP16, SCN1A, GALNT7, CCNE2, R3HDM1, BRWD1, PDE7A, ACVR1, SOCS1, VAT1L, SCN3A, STIM2, KLHL20, LHX1, RNF220, MTDH, STOX2, SETD7, FAM49A, ELMOD2, PPARGC1B, RAB15, TNRC6A, MEX3B, CDH20, RASA1, EVX2, NT5E, CCDC117, ZNF644, DESI2, ATP8A1, PIP4K2A, LGI1, UBN2, SNAI1, MAML1, LIN28B, RARG, LIMCH1, XPR1, NAALADL2, RBM26, EXTL2, CDC37L1, ERLIN1, PRDM1, FAM160B1, PLAGL2, TMEM170B, RRAD, UBE2J1, FZD3, SPEN, STK39, AC005035.1, RAB38, RORA, TTLL7, NUS1, JAKMIP2, PHTF2, JPH4, GLDC, CARF, BNIP3L, MYBL2, DCUN1D3, TENM3, RHEBL1, TMEM181, NR4A2, ADAM19, BAZ2B, CHST2, MBOAT1, SYNGR3, LHX9, ZNRF1, TLE1, CPSF6, SCARA5, SOCS3, PPP1R18, HNRNPUL2, MIER3, USP48, PRLR, GFPT2, CAMK2N1, ST8SIA4, ROR1, MSANTD3-TMEFF1, TMCC1, RUNX2, MAST4, ADRA1D, MAT2A, CUL2, LMBR1L, FOXD1, LPPR4, PROSER1, EPB41, PDSS1, CD99, GMNC, SH3PXD2A, INO80D, KXD1, OMG, TNRC6B, ELOVL5, PEX5L, LRFN2, S100PBP, JOSD1, WDR82, LCOR, LRCH2, AMOTL2, CHL1, UNC5C, RASA2, ANKHD1, ATP2B1, FAM214A, LCLAT1, ANKRA2, BAHD1, ASXL3, RAB32, B3GNT5, DCBLD1, RAB22A, GALNT2, FNDC3A, MMD, MLK4, CBFB, KMT2C, PTGFRN, PAPD4, DLL4, RHOB, ARID4A, TMEM194B, LOX, CEP350, EAF1, SLC38A7, WDR44, PIGA, TAOK1, COL13A1, GALNT1, LRRC8D, MAN1A2, FST, PAWR, CCNT2, TASP1, ZNF521, ACTC1, HTR1F, MFSD6, DPY19L1, PICALM, PCDH17, TMEM87A, TMOD2, LCORL, SEC23A, EFNA3, FAM110B, PPWD1, LIN7C, REV1, SGCB, SEC24A, PLA2G2C, VKORC1L1, GALNT3, YPEL2, TWF1, SNX16, TTBK1, AL137003.1, RAP1B, GNPDA1, YOD1, FAP, SOCS6, NFATC3, MYO5A, RTKN2, UBN1, GCNT2, NFIB, RFX7, ANO4, KLHL2, VPS26B, YTHDC1, SRSF7, OXR1, KLF10, ELL2, CCDC97, GATM, LMLN, RUNX1, XPO1, TENM1, ADRA2A, LARGE, FAM83F, UBE3C, CHST1, MZT1, CNKSR2, LIN28A, ZMYND8, FOSL2, TLL2, sep-08, NOL4L, PHF16, RAB23, HDAC5, UBE2I, SLC30A4, STX2, OTUD6B, TDG, SCML1, SH2B3, AUNIP, ARID5B, IDE, PKHD1, STAC, ZNF280B, MICAL1, ELOVL2, KIF16B, EDEM3, TM4SF20, FAM46A, NR6A1, FAM43A, RTN4R, JDP2, CYYR1, TNIK, ARHGAP26, SMARCD2, TIMP3, CAMK2D, SLC9A8, REEP1, NDEL1, SPEF2, PAPOLB, BDP1, ZNF711, GRM3, PGM3, STK38L, FOXG1, RQCD1, C14orf39, BRD1, KIAA0355, D4S234E, SNX27, ZBTB11, UBAC1, C7orf60, SHOC2, ICK, EPG5, PPP1R2, NHLH2, LIFR, DNAJC13, BNC1, EML1, ANKRD17, SLC6A3, CELF3, DDAH1, TFDP1, GIGYF2, HELZ, PTP4A1, SYPL1, PTPN13, CAPZA1, GPR157, DDI2, ARL4A, SNX18, GOLGA1, AL136219.1, UCP3, E2F7, ZFP1, UBE2V2, RP11-738G5.2, PPP3R1, RAPGEF4, GNAI2, BTBD7, SOX12, MBNL3, MTMR10, PAFAH1B2, PLXNC1, CALCR, ITGA6, PPARGC1A, RARRES1, PAAF1, ATRN, SH3RF1, SP4, PITPNM2, RAB4B, RHD, TM4SF1, TMEM87B, MAST3, CECR2, BCOR, RCOR1, RAPGEF2, CYSLTR1, ATG12, KLF9, DOCK7, ARID4B, ZFY, ABCG5, PPP3CA, CHMP2B, ZBTB6, REEP3, ABL1, ABCD2, AVEN, CALU, CCNJL, PGM1, TBL1XR1, RBM12, DNMT3A, TUSC3, RP11-497E19.2, NFAT5, DDIT4, TBPL1, DPY19L3, SOX9, KDM3A, SAMD4A, LRRC17, PARP8, CBLB, C10orf76, ALG10B, CTTNBP2NL, FAM81A, NAV3, CEP76, C9orf41, WDR70, CADM2, SEC22C, CAPN5, SLC7A11, AVL9, STAG2, NCR3LG1, DPYSL2, GABRB1, RP11-160N1.10, ZBTB18, C11orf21, PNKD, SRGAP3, CPEB3, DBF4, ITGA8, SNX6, RP11-625H11.1, FBXO39, P4HA2, RASGEF1B, LRRCC1, AKAP10, NRXN3, DENND1B, RNF157, ATXN1, SOX4, CLRN1, TRPM7, HDAC9, RABGAP1L, SCN9A, PRUNE2, SOS1, PALM2, TNKS, GRIA2, CEP170, RAVER2, IL1RAPL2, ACTR1A, KCNA4, ZNF197, TMEM86A, PPP1R12A, SSX2IP, PCDH20, EML4, TIA1, STK17B, PHACTR2, RPS6KA2, SLC41A2, CFL2, STRIP1, ITGA4, SNTB2, SPAST, SNX29, HEPHL1, VOPP1, LPCAT2, PNPLA1, FLVCR2, BCL2L11, KCNJ12, RAD23B, MARK1, ARHGEF6, PIK3CD, ATG5, MARCH6, C4orf3, ELAVL2, CAT, BIRC6, RIMBP2, AP4E1, ASB4, ZDHHC21, DIP2C, MIB1, ANKRD20A3, METAP2, HIPK2, FAM133A, FAM26E, USP45, CLOCK, SPOCK3, ANKRD20A2, STT3B, IFNGR2, MINOS1, ACAP2, TTC8, PDE4D, KATNBL1, ago-02, sep-07, ZDHHC14, MAP6, PKNOX2, ELL, ZFAND5, FYCO1, RAI14, STX16, CD2AP, NR5A2, RAB2A, USP37, CAMK4, VENTX, ZSCAN29, TP53TG3, IDH1, SLC6A9, DHX36, ESCO1, NHS, PARP16, TP53TG3C, GNG10, WIPF3, ZNF148, TP53TG3D, RARB, IFNAR2, PGBD2, GJA1, ANKRD20A4, BRAP, PTPDC1, FIGN, CHD9, TTPA, TRPS1, FBXL20, AP2A1, UGT1A8, IBTK, NCAM1, SLC35A3, SLC36A1, AP3S1, SYT9, PDGFC, DLG5, GUCY1A3, TNRC6C, EDC3, PITPNB, GNRHR, ME1, ARL10, EDNRA, PAX3, KLF12, SCYL3, RAP2C, LRRC8B, WDR7, MAP3K2, TVP23C, RECK, ARL6IP6, ZPBP2, USP15, CALB2, FGF20, COL9A3, GNA13, BCL11B, ASB2, HNRNPA2B1, TRPA1, HECTD2, CSNK1A1, AL137145.1, CDK12, BCL6, SLC35F3, FAM13A, ARHGAP29, FXR1, LPP, IKZF2, WDR26, PCMTD2, DSG2, NTM, UBE2D2, SAMD8, MLXIP, RAB21, NAA25, NA, ZFC3H1, SLC1A2, NRIP1, TMEM229A, MBNL1, MYH11, CPE, SEL1L3, CCDC71L, DACT1, SRSF10, SPHKAP, SKIL, IRF4, NUFIP2, VIM, CFHR3, KCTD9, CCDC38, CEP85L, ZRANB2, TVP23B, MYBPC1, MAPK8, DOCK4, TMEM56, SCML2, ORC2, HERC3, SEC22A, LRRC8C, SNX10, PBRM1, NCOR2, MARCH8, GCSAM, C8orf44, DCX, NUCKS1, GAREM, CSAD, CDH13, MEOX2, ATP2B2, WNK3, FAM210B, SPTLC3, C7orf43, C14orf28, C4orf19, CYP24A1, STC1, NEUROD1, PCGF3, CPNE3, FBXO32, PCGF5, WWP1, JAG2, GMEB2, PRRG1, RNF165, KIAA1522, HACE1, LRP6, UNKL, FAM161A, WDFY3, MTTP, MED12L, ANKRD20A1, GPR150, CISD1, BCL9, APPL2, DPF1, EVI5, TRDN, PABPC1L2A, SBF1, AFF4, IL2RA, TANK, IRS2, ERICH3, PER2, SEMA6B, ERG, EPDR1, PPP4R4, TCF21, ANKRD66, DLGAP4, SLC5A3, HOXA11, G3BP1, ABHD13, EMC4, RFX3, ZBTB39, TFPI2, ARID1A, DLGAP2, DSTYK, TET1, SLCO6A1, CCNK, SBK1, SKP2, RUNDC3B, PLXNA2, HOXB8, NLGN1, RAB7A, FNIP2, TSEN15, SLC35C1, SNAPIN, FAM24A, SPAG9, SSBP2, C1orf174, TULP4, TRIQK, IRS1, OVOL1, BNC2, NKX2-2, SNX33, SESTD1, ZNF720, PTPN20B, CBX8, LRRC40, PRKRIR, SLC22A5, FOXP2, NRBF2, MYOZ2, NCK2, ATG2B, SIRT1, ZNF704, USP22, DOC2A, ALG9, BSN, MCTP2, ITGB1, RAB11A, KIAA1147, PVRL3, BTNL2, MNT, AFAP1L2, NDUFC2, RFTN2, LYN, MIA3, AP1S2, IFNLR1, PLAG1, FGD6, NEURL1B, GPCPD1, CNGB3, EPHB2, C7orf31, ABCC9, ARID3A, MAP7D3, CNTN4, DMD, BMI1, FBXO28, LRRK2, KLF8, MCF2L, TMEM26, HIC2, DDX5, GULP1, ADAMTS3, EPC1, ALPK3, VAT1, LONRF1, PLEKHO2, ATL2, RRM2, NFATC2, GATA5, COMMD2, PANK3, WIPF1, PRG4, VAMP3, TCF24, DOK5, CCDC171, DOLPP1, SDAD1, DDX46, PDE5A, DAB1, NAPG, C3orf14, SFXN1, KIAA1244, ABCA12, GSKIP, PTPN20A, CTC-241N9.1, PTPN4, UGT2A3, NECAP1, PRSS37, EIF4E, GOLGA4, CYB561, RPL26, SIM1, AP1B1, RBFOX1, TNKS2, TOX, GAGE1, SLC35G2, PDGFA, ZMYM2, PABPC1L2B, NUP93, LIMS1, CSMD3, NEFM, SSR3, RMDN2, FBXO45, ZNF805, POP1, SLC10A7, DENND2C, CEACAM1, KLF7, CLCF1, YPEL5, ITSN2, VPS13C, FAM199X, EIF2S3L, ADAM18, FAM189A2, ITPK1, UBE2R2, GXYLT1, GK5, OTUD4, ATAD2B, MAP3K5, GNAQ, RASGRP3, DMXL2, EEPD1, GLT1D1, PPAPDC2, EPB41L5, RAB3D, SYBU, AC104472.1, CNOT6, MAGI2, BECN1, MICB, ZNF831, CDCA7, TPM4, RCBTB1, RALGPS1, PSTK, MEIS2, MSS51, OSBPL8, FLVCR1, GFM1, CCDC144NL, ZDHHC17, SGMS2, PIRT, GLCE, SNX8, KIAA1024, TNFRSF10B, NUDT5, FAM169B, SLC35F1, ADAM12, EFR3A, VAV3, AMOTL1, KCTD7, ONECUT2, NECAB1, ZNF529, USP2, CERS6, CCDC50, PLA2G12A, HLX, ZNF264, ATF1, CSNK2A2, ABL2, TPP2, PNMA1, NAGPA, CPEB2, FAM217B, MIER2, DHRS4, REV3L, HORMAD1, KCTD5, GPR139, RANBP2, ENTHD2, UBE2F, ITGA9, ELMO1, CPEB4, LHFPL2, SLC26A11, ADAMTS9, KCNIP4, RAB8A, TMEM47, CACHD1, FAM9C, ZNF594, FAM73B, NOVA1, MORF4L1, ZCCHC11, SEMA6D, PPP3CB, MFSD11, UBE2G1, CASP3, GRIN2A, NEGR1, AC011897.1, HECW2, MAFG, FMR1NB, FYN, PPTC7, KIAA1715, MAGEE1, ZNF547, FBXO34, AR, BEST4, ago-01, BAZ1A, ACTN1, ZDHHC2, C3orf58, B4GALT4, SLC4A7, RANBP9, FRMD4A, JMJD1C, TMEM170A, SLAIN1, KMT2A, DSCC1, ASAP1, VIL1, DNASE2B, GTF2E2, ENPEP, KCTD3, NADK, MARCH4, MAP3K13, NEDD4L, SEC23IP, ZNF585A, CBX2, TOPBP1, MOB3B, NR3C1, IQCB1, LMO3, RFX2, PDE4DIP, FOXO3, POLR1A, TDRP, SEC61A2, YWHAZ, LPGAT1, SCAF4, CTPS1, TMTC1, ZBTB7A, EYS, KIAA1328, RSL24D1, NLGN4Y, PPIP5K2, GCLC, C6ORF174, TRIM37, AZIN1, SLC36A4, FOXN2, AMER1, ERC2, C6ORF174, CACNB2, C3orf38, PREP, PITPNC1, DAPP1, ADRB2, FBN1, TMPRSS7, MEF2D, PSD3, ORC4, RGL1, ZYG11B, TOMM70A, PPP2R1B, SOGA1, ELK3, TTN, GRM6, CUL3, C16orf87, FBXL17, M1AP, EIF5A2, CNOT2, NID1, TM9SF3, PPIL4, ADAM22, TNPO1, ZNF625-ZNF20, KRAS, PNISR, SLC35B4, POLR3G, ARFIP1, ATP11B, SOX13, RRP1B, CCNA1, NACC2, TP53INP1, FAHD1, MARK3, SMAD2, KLF13, EDAR, TWISTNB, TARSL2, KIAA1279, MXRA5, C10orf25, APC, YBX3, ATP8B1, EEF1A1, DCTN4, INHBA, TK2, USP44, C6orf201, SLC9A4, ZSWIM6, PPIL2, LYPLAL1, PPHLN1, CLASP2, SLC25A36, UBE2D1, GPR108, CCDC14, RNF213, YY2, KIDINS220, RAB27B, SPCS3, PPP2R2B, MECP2, EMCN, ZBED1, FAM165A, BET1, NAA35, HIPK1, BCL10, NEDD4, NRBP1, VAPA, FLT1, ZIC1, JARID2, FMNL2, FRZB, DCAF17, TRMT5, LARP4, WDR1, VGLL3, FANCF, IGF1R, PAQR3, SUPT3H, TRIM9, IER5, PPP1R14C, ATL3, GABRA5, STEAP1, MMP21, ATP2A2, RASAL2, C8orf34, MSANTD4, C16orf52, MAP3K7, RNASEH1, GDNF, KIAA1432, TICAM1, DACH2, KIAA0226L, PTBP3, GPATCH2L, VCPKMT, EZH2, FAF2, PTCH1, GRK5, SGSM1, IRF2BP2, ARL8B, DGKZ, GRB10, DGKH, NCOA3, KLHL4, STYX, SLC38A2, TRO, CNR1, POLR1D, ENOX2, RCHY1, TMEM35, STOML1, MFHAS1, HECW1, RNF121, TMEM245, PSTPIP2, USP14, ZNF141, CDH11, DCUN1D1, RNF217, AIDA, RAD9B, FAM109A, BRD3, DTNA, KCNJ3, ERBB4, ESPN, NFYB, ABI3BP, EPHA5, RNMT, SORBS2, CDC14A, TRIM13, CHRM3, CTHRC1, NR1D2, SHISA3, SKIDA1, HMGB3, NOTCH2, TACC1, CHFR, SUV39H2, DNAJC25-GNG10, P4HA1, C3orf65, TAB3, PPIL3, N4BP2, ANXA11, LATS2, COPS2, ACBD5, PTAR1, CCDC148, PABPC4L, ELK4, SLC22A23, ATAD5, RALGPS2, TBC1D30, RIOK3, KPNA3, GPT2, LCP2, KCTD8, KDM6B, HNRNPA1, TSPAN33, TMEM246, EIF1AY, FBXL14, ZNF770, PLS1, CEP44, EPHA3, NR2F2, KIAA0319, PIGX, SGK3, HCN1, NAP1L5, PIP4K2B, VIMP, FUCA1, GRM5, RPL37, ZNF680, SNX30, TGIF1, SNX1, PEG10, KIF3A, STAG1, MYO1H, ZNF333, LYRM7, MSI2, KIAA0284, SPTY2D1, RPRD1A, BLOC1S5-TXNDC5, NXT2, IGF2R, SIX1, NR3C2, CA10, SERINC3, FAM126A, ASAP2, CAND1, KSR1, ZFX, CRISPLD1, KIAA0368, ITSN1, SLX4IP, NCALD, ATG4A, EBF2, ANKS4B, PSME4, SLC6A6, CATSPERG, PRRT2, CHORDC1, SPA17, ZNF367, KLHL7, PRKAA2, PELO, B4GALT6, SLC44A3, MRPL50, TRIM59, PPFIA2, KLHL15, PROX1, ABCC10, DIP2B, AP3M1, TSPYL4, DHX40, CELF1, CAPN7, BEND7, RSRP1, HIAT1, ATP6V1B2, FAM84A, LHX5, KMT2D, ZNF507, KIF11, SNAPC3, AASDHPPT, EXD2, ZNF510, PARP15, MUC20, HBS1L, PRICKLE1, PLA2R1, TRAPPC6B, PTK7, COBL, PLCG1, NUPL1, AC012215.1, MICA, SLC2A14, HCFC2, TRPM1, IYD, CACNB4, PRMT6, FGL2, HTR4, RIMS1, SALL4, TMEM161B, GLUD1, EN2, SLC29A3, CYB5B, SLC39A10, BARD1, CNTN1, SLC30A8, RC3H2, RPGRIP1L, CBX3, NSUN3, METTL20, BCL2, SMURF2, ZCRB1, USP47, PQLC3, C9orf85, LRP12, BACH1, BRINP3, KLF11, PTPN21, TRIM35, UGT8, ESF1, ELAVL3, POU4F2, RPA2, RRAS2, LCP1, ITPR1, KHNYN, STS, RGCC, MBNL2, PTEN, LPHN3, PELI1, CKAP4, ITGA2, RNF168, RTCB, OR11A1, C4orf17, GPBP1, ZNF100, RASGEF1A, KLF3, KDELC2, GALNT13, FAM46C, UBE2D3, GTF3C4, RPS6KC1, ATP8B2, ATXN7L1, ZCCHC14, UBE2K, LONRF3, HOXA1, PPP1R9A, MESDC2, IL1A, CEP120, INSIG2, RBM15B, KREMEN1, SOX11, BOD1L1, ARHGEF12, SIDT2, EYA2, CECR6, TOMM20, MED6, UHRF2, TMED10, CERS3, TPH2, UQCRB, ZCCHC3, SIX4, ALG1, HGF, GAS2, MPZL3, C21orf91, NF1, C10orf90, BID, DHRS4L2, AKAP6, JAM2, STXBP1, MEX3D, SV2B, RFFL, USH2A, SNAI2, CDHR3, VPS13A, SDK2, PTGER3, ARHGEF40, IFNA21, SLC35A5, ACSM3, CARS, HDGFRP3, GTF2H1, EDIL3, SMIM14, FN1, PATL1, PDP2, ACP2, GUCY1B3, TECPR1, TMEM121, CCL19, STK35, TMEM110, RRN3, SLAMF1, PHF20, HOXA13, UNC13C, PDCD10, ARPC5, ROCK2, KRTAP26-1, IPMK, GTPBP10, GATA6, NRK, TBC1D2B, ERCC3, TMEM33, CRB1, SIKE1, C9orf84, LRRC58, AMER2, NKAIN2, PIK3C3, LYSMD3, TBC1D15, PARP6, UACA, ZDHHC15, FRMD4B, FAM156B, NEBL, MTBP, KIAA1211L, ATP7A, CHIC1, DACH1, BOD1L2, VPS41, ZHX1, YTHDF2, FHOD3, GM2A, LRCH3, DENND4C, TMEM255A, PRPF39, LPAR3, TNFSF9, GOLGA8A, NEUROG1, CREG1, NOL12, PCDH9, UHRF1BP1, U2SURP, ANKRD22, HIPK3, FOXA1, C15orf40, MARCKS, PDS5A, ZBTB38, ANAPC1, CSMD1, CHI3L1, GRK6, TRAPPC8, JUNB, SAR1B, TMEM67, FBXW7, SORCS3, ZFHX3, SMDT1, NTNG1, WDR19, TNIP1, PGM2L1, SLC16A14, EFCAB14, FPGT-TNNI3K, GPR124, ZEB2, TNFRSF10D, MTMR12, SGCD, ZNF275, COTL1, CLCA2, MFN1, PLXNA1, UNC5D, ZCCHC10, ARHGAP28, AKIRIN1, RNF103, MIS12, ECT2L, DCUN1D4, CTH, PDE3A, AFF3, SURF4, FAM156A, FAM104A, BCAP29, NEK4, WDR75, SYNC, CCNF, MFSD8, C19orf12, TM4SF18, NBPF20, AHR, CD80, SSH2, DYNLT3, COL4A3BP, FUBP3, SOS2, FAM115C, PHC3, ITGA5, FUT4, WASL, RASGRF2, DYRK1A, COG2, GPRIN2, LTN1, C10orf126, SEC31A, HTRA3, PHAX, RWDD4, HAPLN1, MYH10, BLM, RPGRIP1, JAKMIP3, SEMA6A, INTU, ZNF268, FAM109B, PPIG, CXorf23, FRMD6, DDHD2, IPO7, WDR43, N6AMT1, CEP78, TSPAN8, NTRK3, SORBS1, ANLN, KBTBD7, ANKRD31, SETD2, TRPC6, PAPD5, PDS5B, MON2, PRDM13, PWWP2A, ZNF318, DNM3, PSMC6, ELFN2, SLC39A6, TMED2, DERL1, RAG1, ZNF451, FRS2, ZDBF2, ZNF621, MAP3K1, RASSF8, P2RY10, PIK3CA, ZNF566, PM20D2, ZRANB3, SLC12A5, GRHL2, PIK3R2, EXOSC3, RAB14, NOTCH1, STXBP6, ZNF491, TBC1D8B, FCN1, OSBPL3, UPF2, YBX1, MYD88, PAK7, PHLPP1, KIAA0087, ZNF200, SEH1L, CD6, UBQLN4, POM121, CALML4, PHF3, LRRC19, CYCS, HSPA14, RGS1, PRKAA1, EEA1, IRAK1BP1, SLC30A1, HLF, UHMK1, CCDC18, WAPAL, CREB1, MAPRE1, MAGI3, URM1, USP9X, LOH12CR2, ZC3H8, RECQL |
| miR-92a | BCAT2, DKK3, KLF4, UBE2W, MAST4, DSCAML1, ITGA5, TEF, FXR1, WASL, ARHGEF17, ARRDC3, MYO1B, GRAMD1B, SGK3, FAAH2, GOLGA3, CD69, SOX4, BCL11B, PDZD2, KLHDC10, PCDH11Y, PTAR1, BAZ2B, HIPK3, MAN2A1, PCDH11X, ST6GAL2, FNIP1, ADAMTSL1, ERGIC2, MYCBP2, SLC12A5, FMN2, MIA3, CAMK2A, CIC, CPEB3, TGIF1, FBXW7, MAP2K4, PPP1R37, RGS3, FNIP2, NKX2-3, BSDC1, NOX4, C2CD4C, SFXN1, LHFPL2, DOCK5, POLK, DENND4B, TCF21, SNN, DUSP10, BTG2, GOLGA4, RNF44, TAGAP, CDC27, FMR1, EVX2, GRHL1, TOB1, RNF38, PDE10A, MFHAS1, KIAA1432, RAD21, JMY, SLC17A6, ADCY3, UBE2Z, ITGAV, PLEKHB2, FAM20C, KIAA1279, CNEP1R1, ADAM19, PGAM1, NEFH, SIK1, RAB3C, PPP1R12A, PNISR, SNAPC1, OAZ3, EDEM1, ACTC1, GPR180, FAM110C, MAP1B, EVI5, MYLIP, CPEB2, FZD10, ROBO2, GRAMD3, COL27A1, FNDC3B, SLC7A11, MMD, GFPT2, RBM47, ZNF287, SPRYD4, REST, ATXN3, DMXL1, PPCS, TMF1, PDZD8, FAM150B, PCMTD1, PTGER4, ADAMTSL3, OTUD3, CNIH1, SH3PXD2A, ATP8B1, CUX1, SLC24A3, SESN3, TMEM255A, REXO1, TEAD1, DUSP5, IDH1, ARMC1, DSC2, MSRB3, LATS2, GAA, PITPNA, EFR3A, NFIA, NOVA1, DNAJB12, SELT, KCNC4, SLC38A11, CHST1, TEX2, APPL2, CACNA1I, ZNF230, ASPN, CCNJL, G3BP2, NECAP1, NEFM, ZFYVE21, DLGAP2, PLEKHA1, PPP1R12C, SIK2, RNF141, SHOX, TMEM229A, RORA, ATRX, SGPP1, TOB2, RAB23, ATP2A2, ARL5B, GOLGA1, STRN3, EPG5, SH3D19, DSTYK, BAHCC1, PTPRO, PAPD7, GDF11, CBLN4, CTTNBP2, AARS, ESRP1, RGS17, KLHL14, IRS2, DDX3X, OTUD4, HERPUD2, CLDN11, IKZF4, PIK3CB, ZNF804A, FHL2, PITPNM2, SPOCK2, SLC9A2, ZBTB10, TULP4, GPR137C, CSMD3, FAM19A1, SLC4A10, WWP2, PIK3R3, MEF2D, KIAA1024, TRAM2, MTDH, AHCYL1, B3GALT2, CPEB4, ACOX1, OSGIN2, RNF11, RBM27, PIK3AP1, RSBN1, RBFOX2, FOXN3, SSFA2, LUZP1, ATP7A, RAG1, CCT6A, DYRK2, RABGAP1L, HCN2, PHLPP2, STYX, BSN, DENND1B, SLC6A1, LMBR1L, REV3L, SLX4, FAM160B1, JOSD1, KLF2, SBNO1, NFIB, WDFY3, HNF1B, PIP5K1C, FCHO2, FBXO33, PLEKHG3, FAM24A, CALN1, ITSN2, PTPRG, C21orf91, SYNJ1, GPBP1L1, KIAA1109, GOLGA8A, PTEN, CDK5R1, AP000295.9, USP34, JPH2, LRRC69, TSC1, PIK3CA, PHTF1, DNAJB9, NLK, S1PR1, TMEM143, BPY2, SOCS6, ANO8, NFYC, AC012123.1, CDCA7L, PER2, BPY2B, CCNC, BPY2C, COL1A2, ABHD13, HAS3, CD2AP, ASXL2, DNAJC27, DAAM1, DYNLT3, PPP1R9A, INSIG1, SLC52A1, RBPJ, LRRC1, MARK1, HES2, ITPR1, PITPNC1, NIPBL, RAP1B, PALLD, SLC5A7, FRY, EIF4G2, CCSER2, NKX2-4, SCRG1, CHMP7, FAM135A, CHKA, GIT2, AP1AR, HMX3, KLRF1, SYT1, VASH2, JADE1, PDS5B, KLHL15, ZNF721, TRIM6, KCNA1, BCAT1, SLC30A7, BMPR2, PMEPA1, PCDH9, PAPOLA, CXXC4, LDLRAD4, USP45, FAM133B, SUDS3, ZNF726, KIF5B, ROBO1, MCOLN2, DST, PAX3, CCDC186, EXOC3, NUTF2, NR4A3, TMEM87A, PDE8A, DPP10, VPS54, ZFHX3, RNF4, SERTAD2, DAB2IP, XYLT2, CSMD1, CEP350, EIF1, DDX3Y, TRAK2, PRDM16, SOX11, L3MBTL4, COL5A1, SLC9A1, LIMCH1, NEUROD1, CADM2, ZNF530, ITGA8, GLTSCR1L, ZSCAN5B, NRXN3, AXL, FOXP1, ANKIB1, PRDX5, UPF2, FAM81A, KLHL29, SEL1L3, TBC1D19, FOXN2, ACADL, PTPRD, ADM, RUNX1T1, AURKA, PAX9, GOLGA8B, RRBP1, ADRB1, GPR111, TBL1XR1, ZAK, BTLA, LRP1B, LANCL3, MTF2, HAS2, ZNF532, CHST7, FBXO28, MYO5A, ZNF24, RBFOX1, TNS3, ZNF521, PAPOLB, APH1B, HOXD10, SLC12A2, RP11-315O6.2, AKAP10, MOB3A, PRDM15, FNBP4, MORC3, SETD2, AFF1, MTHFD2, SFTA3, SLCO1B7, SNAP91, TMX4, RFX1, RNF180, ZNF654, SERTAD3, NFIX, EPS8, ENPP3, RANBP9, KPNA5, USF2, EXOC5, COG3, LEPREL2, INSL4, GATA2, ZBTB18, ASAP1, TPCN1, FRS2, CHRM5, SORL1, SMIM11, SLC2A3, PIAS4, NSMF, KIAA1671, MOAP1, SEC24A, PPARGC1B, GOLGA8F, ANO3, CCDC89, MAGEC2, SART3, MYH3, C5orf28, SLC2A14, TAF1, FAM214A, PIKFYVE, SATB2, RP11-861L17.3, FBN2, SMURF1, RNF157, RP11-113D6.10, SLC39A8, YIPF4, KMO, FRYL, ITGA6, UBE2G1, ARHGEF10, PALM2, KSR2, ANP32E, ZNF667, SLC16A14, MARCH4, SVIP, INSL5, PPP4R2, SPTBN4, NRK, SREK1IP1, SLC16A6, CDK16, FKBP1A, ZNF41, SLC25A45, PPARG, ARNTL2, ATL3, ANKRD28, TBC1D12, PFKM, HECW1, GATA6, MIER3, EN2, PAPD5, C9orf85, CCDC113, SLC30A4, TCF4, VSIG10, SSBP2, PCDH10, SIM2, PALM2-AKAP2, FBXL17, SFMBT2, SLC6A17, MFSD1, TMPRSS11B, TMCC3, GOLGA8IP, ZNF385D, ADO, FBXO32, TP63, FRZB, CASD1, GPR158, SIK3, MMP16, H3F3B, KLF12, NOTCH1, RP11-507M3.1, BX255923.1, ATXN1, CDC42BPA, ZFC3H1, TMSB4Y, PFKFB4, EZH2, TNRC6B, ZNF140, RNF217, UNC79, PDCD6IP, MTMR9, TTC28, VPS36, LRRC20, MRPL17, GAN, C2orf69, ZNF827, FST, NAAA, GOLGA7, CNTN4, EGR2, ARHGAP31, MSR1, FAM221B, ZNF253, NIPAL1, HOXC12, PAIP1, ADAM10, RAB8B, COL19A1, PDXDC1, OSBPL9, IFIT2, ABI3BP, MKRN2, PEPD, ATP11C, PKP4, BICC1, MAP4K5, VEZT, SLC11A2, TLE1, DNAJC2, NSMAF, UBR1, CPSF6, ZNF512B, DPP8, TMOD2, GRIA1, PIP4K2C, HAND1, FAR1, PRDM13, TRAF3, HAND2, NAA30, SYN3, KCNJ3, MARCH8, PAPSS2, GLYR1, ZNF813, LATS1, TOP1, PUS7L, WNT5A, KRT1, CAND1, RP11-156P1.2, VWA5B2, TRIP13, GRK5, FASLG, PSMA8, DDIT4, PATE4, TM2D2, GPR85, KY, TRIM33, GNAQ, STRN, DESI1, SNAI2, PLEKHG1, MEGF10, TRIM36, AFF3, RPE65, NAGA, ZNF264, CAPRIN2, FHL3, LPPR4, SLC30A8, ASB5, CLK3, KBTBD8, DIRAS1, SYNDIG1, ARF1, BCL2L11, MLLT10, SWT1, ZFHX4, NCOA6, ALX4, GATAD2B, WDR81, ELOVL4, FAM196A, ABCA1, C1QTNF3-AMACR, FAM227A, SRPK2, SORCS3, UBASH3B, JADE2, PDE4D, FUNDC1, FOSL2, PHTF2, ITCH, GLUL, ATG14, DDI2, ZBTB34, RAP1A, CYYR1, RBM24, BAI3, BCL9, TMTC4, CLEC16A, SRPR, SLC25A32, PAPD4, CLCN5, PRRC2B, N4BP2, STK39, SLFN5, PAX5, NCOA1, TCTEX1D1, ESCO1, HEG1, SDPR, TRIP11, KIF3B, MFF, GSK3B, MPP1, PIGA, MAP10, ARRDC4, CBFA2T3, LPIN1, UHRF1BP1, ATP2B4, DACT1, CXXC5, SKOR1, PRKAR2B, SCUBE3, MTPN, IGFBP7, KDM2A, PNLIPRP3, SETD7, FCRL2, NPTX1, CREM, MITF, ING2, MDGA1, CABS1, RECK, TTLL7, ZDHHC5, GLCE, SMIM5, STEAP2, AC011294.3, SIRT6, KLF3, GRID2, RP11-770J1.4, DUSP28, RASSF3, TECPR2, RPGRIP1L, ZNF492, ASB7, KLRD1, TNFAIP6, KCND2, USP44, IRF1, PVRL1, COL12A1, IQGAP2, CDC42, GCLC, PI15, MRS2, SLC26A2, TMC8, ELOVL6, COX20, EEA1, CES5A, SLC6A14, GRM7, OSER1, CEND1, RAB11FIP1, ANKRD13C, SAR1B, ZFPM2, VEZF1, PDHB, TNKS, EPHA8, LRRC8D, ACSL6, ANGPTL2, XPNPEP3, NUP43, PRSS12, CD59, LCOR, AP3B1, DCLK2, LRRC4, AFF4, COPS2, NHLH2, EDEM3, NEURL4, TMEM87B, SLC38A2, MAP3K2, PARD3B, ADAM18, HNRNPA3, SLC4A7, CXADR, PLEKHM1, PRPF40A, CASP16, ADAMTS9, IKZF2, SP6, ZNF469, XRN1, DCUN1D4, MICU1, MTMR7, GOLGA8G, RWDD4, SYNE1, IRG1, CUL3, DCAF17, ARPP19, PCDH18, PTGES2, FBXO47, ARID5B, MYH9, LBX1, CTNNBIP1, PCGF3, C7orf25, HERC3, MOCS2, RRN3, FAM46A, RNF2, TMCC1, SOSTDC1, ESRRG, ZFP37, ELFN2, SDC2, MTRNR2L5, PDE1A, STS, HERC4, TIA1, PRR24, MPZL2, SIM1, ZBTB21, CCNE2, ABL2, KAT2B, G2E3, CXorf24, TENM1, FAM76B, CHD9, ANO4, LGI2, PCYT1B, PPIP5K2, FAM110B, TBX15, SUV420H1, NKPD1, TMPO, HOXC8, HIPK1, VAX1, BCL11A, EIF2A, SMAD7, RYR3, LIN54, NOL4L, ago-04, RAB30, CXorf57, ZHX1, C5orf24, GCM1, CDKN1C, WWC1, RAP2C, TGFB2, SLC9A3R2, PDGFD, FGF5, CALM3, RP11-766F14.2, GRIA4, PRKAB2, CRB1, SPEF2, MYNN, DTX2, WDR20, GUCY1A3, CSF2RB, ZNF124, ABI1, TMED5, PTCHD3, PUS7, TWIST1, PLCB1, CEP162, TNPO1, ZNF385B, SHPRH, PCDH20, FRMPD3, DCP2, CNNM4, BPHL, RASAL2, GHR, CLGN, ATP11A, DCX, SLC35F4, SOCS5, C9orf47, AACS, SF1, ELK4, TBC1D4, ST3GAL2, AADACL3, NCBP1, NPHP3-ACAD11, SMARCAD1, SLC46A3, TEK, LRRC8B, MAP3K13, GART, CACNA2D1, PEAK1, MTMR10, NFAT5, ICK, ERBB2IP, RFX7, NCOA3, AQP8, SLC25A31, ARHGAP5, DAG1, DCAF8L1, HP1BP3, GID4, FAM117B, ADRA1A, VAMP2, PEX3, SLC5A12, CREB5, NAA25, PCGF6, MED13, USP9X, METTL7A, GAA, FOPNL, GCNT3, FARP1, EMR2, C14orf28, GCLM, ANO10, UBE2Q2, MOB3B, ABI2, NRL, KLF6, NHLRC2, RGL1, KCNC2, CCNG1, EGFR, MBLAC2, GAP43, TMEM74, PTGFRN, ZNF273, TGFB1I1, MBOAT2, ZNF850, MYO1C, HNRNPA1, FAM160A2, DKFZP686D09174, TMEM215, FBXW11, NDUFC2, FGD5, C1orf21, RP11-455G16.1, RAD51B, MYT1L, NEGR1, TMEM154, PVR, TMEM242, DISC1, STK16, GIPC2, NPTN, CEP57L1, ADAM23, ZNF772, ZNF449, CRHBP, GEN1, HMGA2, ATOX1, LRRN1, SCARB2, PRKAR1B, FKBP1C, MKL2, MYO18A, ZNF585B, DUSP1, TAX1BP1, CPE, CHGA, NCOA2, SLC45A3, CGRRF1, PPP1R1C, SGK1, SEMA3A, NFATC2IP, CCDC67, AC104472.1, KLHL21, DMRT1, IGSF10, SMIM10, DPPA4, MDM2, ZBTB40, MGLL, TRPM3, HBS1L, FAM126B, TTC26, ANKRA2, PARP8, PDGFRA, EYA4, ZNF286B, LAMP2, C20orf194, PRKAA2, MAP7D3, SMAD2, SNAP47, SRXN1, ZBTB46, MBD5, LCE1E, ERC2, ADGB, NICN1, COL11A1, TMEM232, FRAT1, CENPP, RPS6KB1, FAM84A, EPM2AIP1, TCTN3, ZNF35, FNTA, PROX1, MIPOL1, ABLIM2, PRKAA1, FAM156B, NAV3, UGGT1, ADAMTS3, SLC1A2, IL12RB1, NRF1, NEDD4L, METAP1, ADAMTS5, CCNJ, MSRB2, GRAMD4, EZH1, SPATA18, AC006455.1, SH2D4B, RAB39B, TRHDE, NAT8, STAG2, APPL1, RAB14, BACH1, RICTOR, JRKL, RREB1, SSH1, ZNF805, GABRA3, TTC19, COL4A3BP, JDP2, CACNA1C, ALDH6A1, TPMT, PEX5, CHSY3, GLIPR1, FBXO21, ZNF148, RPGR, PANX1, DACT3, PPP1R13B, PDCD10, SLC39A14, CCDC144A, TCF24, LIN28B, CREB1, C11orf72, KMT2E, SS18, P2RY13, GRIA3, NPNT, PTF1A, C16orf87, AHRR, RUFY1, CTDSPL, YWHAH, ATG3, CACNA1H, GNB3, ALPK3, CCND2, CREB3L2, RXRB, SEC24C, TMBIM4, NXPE3, KIAA1377, RAB6A, IKZF1, EBAG9, PRND, METTL10, HGF, ITGB6, TTC9, CLUL1, ZNF516, PIK3R1, VWA9, TMC1, DMRTC1, AZIN1, HELZ, NEK7, CD109, CASP9, HNRNPA0, C16orf72, AIDA, APOBEC3F, NFYB, SPINK5, ZNF770, KCNMB2, NEO1, VCL, NRXN1, DPY19L4, LRP10, DYRK1A, VENTX, ACVRL1, PTAFR, CAMK1D, BDNF, UACA, IL7, PAQR3, NMNAT2, HIBADH, ENTPD1, RNF10, TAF8, ABCD2, FAM105A, ABCF2, GIPC1, ARL2BP, ACAP2, MSANTD1, ABCG4, NAA35, GATM, |
| miR-125b | FAM131B, NBPF10, ZSWIM5, PCTP, SEMA4D, KNSTRN, KIAA1522, SMEK1, FUT4, TNRC6A, LFNG, ENPP1, TSNARE1, MFHAS1, DRAM2, NPL, USP2, SUV39H1, ENPEP, ZSWIM6, TRIM71, ZNF543, C6orf47, RBM20, GJC3, SLC46A3, FAM169B, MYT1, BAK1, NUP210, ZSCAN29, STARD13, MAP3K11, ARID3B, CRB2, SLC35A4, ABHD6, FAM27C, KLF13, MCL1, GCNT1, ESRRA, NCR3LG1, LBH, FBXW4, LRFN2, ORC2, USP6, KCNK10, TTPA, WARS, DUS1L, ZFYVE1, RFX5, CGN, C19orf38, PDZD3, PSTPIP2, TGOLN2, GTF3C3, SLC39A9, GGA2, MAP3K13, SSTR3, JMJD1C, TRIAP1, GTPBP2, SLC4A10, UBN1, FAM134A, SLITRK6, SMG1, CCNJ, TMTC2, TBC1D1, CPSF6, DAAM1, PRTG, EVA1A, CORO2A, ATP10D, SBNO1, FPGT-TNNI3K, LRRC10B, CYP24A1, PPAT, SEMA4F, DYRK2, OSBPL9, SYVN1, LIN28A, KLC2, NBPF20, ZNF704, CCNF, NBPF16, AC009237.1, TMPRSS13, NEU1, MINK1, MASP1, CTTNBP2, DDB2, INO80D, C11orf57, MAP4K2, AIFM1, ADPRH, SEMA4C, GANC, ORC5, FPR3, ZFP62, TLL1, IL16, CHTF8, IRF4, ZNF792, VPS4B, FAM27A, SAMD10, INTS7, VAX1, CDC37L1, SLC25A15, SLC35G1, IER3IP1, TOR2A, RORA, CYTH1, KLHL31, KLHL6, JADE2, BIN2, TRPS1, TNFAIP3, SH3TC2, PRDM1, VNN3, HCN3, KCNS3, ZNF281, MLF2, CARM1, IST1, PCSK7, PTH1R, ZNF827, TET2, CD34, KIAA1841, CSPP1, SCARB1, IGF2BP3, UBE2D3, NRXN1, TRMT5, ZNF839, LRP4, BRMS1, DHX33, MYLK, DIP2A, SLC17A7, TMEM168, RAPGEFL1, ZNF460, MAP3K10, LRRC69, FAM118A, WI2-3658N16.1, GGT7, TAF4B, RABEP2, GSTM2, FAM205B, TAF12, SLC7A1, ALPK3, ANKRD50, ZNF691, PKP4, C19orf54, KCNJ9, CCR5, MRPL30, C17orf59, IBA57, CELF2-AS1, TAF9B, KIF15, KPNA6, KCNIP3, DPY30, BRWD1, NLRC5, DOCK3, FLT3, GLYR1, ACAD8, LCORL, IL6R, NAIF1, PMM2, TBX2, C17orf51, SPEG, ITGA8, RNF168, SECISBP2, BRPF1, XKRX, MEGF8, NBEAL2, SCARB2, NECAB3, ST6GALNAC6, ARID3A, RS1, RASGRF2, C2orf43, NT5DC1, RAD51C, KMT2C, IKZF3, NLRP1, MBD1, ABTB1, TCTA, FRMD5, SIM1, SWSAP1, RYBP, UBE2R2, RMND5A, CSNK2A1, NBPF9, GRB10, TMPRSS4, KDM7A, PTPN18, ACSS2, TMEM101, TLK1, EPO, KHNYN, BMF, CHD7, ZNF236, CXCL13, SLC26A6, ZNF385A, DPH2, ZNF774, LIFR, MCTP2, NBPF12, NBPF14, NR6A1, OLFML2A, CMTM1, NBPF24, TLE3, CDR2L, ST6GAL1, C10orf54, MAPKBP1, RAB3D, TRAF6, PYHIN1, THEMIS2, LCOR, BLZF1, MAP2K7, PHACTR3, SESTD1, CDH13, VTCN1, ARMC7, DDX5, RREB1, ZC3H7B, STXBP5L, SH3BP5L, GPR107, DAZAP2, MXD4, SERTAD3, TNFSF4, SOX30, C2orf15, ETV3, MEGF9, HOMEZ, ARSI, ADAMTS14, DIRAS1, PARP8, ITGA9, CDC42BPG, DYNLT3, TMEM2, CTU1, LYVE1, SRRM3, ADAM28, RIT1, BCAN, ZNF624, RUSC2, MFN1, MAPK12, RTN2, GPATCH2L, SAMD14, MGAT4A, FAM65B, CNTROB, NBPF15, TAP2, CDK16, ELP6, SEL1L, SIT1, KCNA1, KCNA7, LIPA, EBF4, GJC1, LNPEP, AMER2, AC015804.1, ERMP1, TDG, APLP2, ELAVL4, BDH1, ZNF343, FOXD2, SMURF1, XPO1, TMEM194A, LCLAT1, CTD-2021H9.3, USP7, INTS6, NXF1, ZNF831, RP4-539M6.19, KLHL7, C4orf17, KSR2, TOMM40, TMLHE, MBOAT2, DHX30, CD48, TRPM1, PIK3CB, TP53INP1, SLFN5, ATL2, BAP1, FBXL5, CTNND2, LIN28B, TMEM120B, PODXL, PAFAH1B1, PPM1H, PSG4, FAM107B, ABCC5, NIPA1, PTPN1, GPR153, SCARA5, CHAMP1, VPS37C, HINFP, STAT3, CCL4L1, RALGPS2, RFC5, UBE2G1, ARHGEF2, SOX11, AC013269.5, SZRD1, VWA3A, AMIGO2, ETS1, ZSWIM4, BBS4, LRRC8B, GLS, FREM1, TSTA3, KCTD15, ULBP1, BTN2A2, MAP3K1, PRAMEF18, TMEM63A, ATHL1, KCNC3, ROBO4, DTX4, BCL2L14, ABCC2, FLG, ICOSLG, IKZF4, ago-03, NBPF11, PGAP3, SCRT2, PSMB8, PRAMEF3, PLAGL1, PHC2, SETD6, BCAT1, HOXB3, E2F2, JARID2, CSNK2A2, PPME1, MAPRE2, SUZ12, MSRB3, SSX6, SATB2, BAZ2A, PARM1, ENTPD4, MSI1, TMEM180, FMNL3, NIPAL4, NFATC1, C1orf147, BRCA1, LEPROT, CCNJL, ZBTB7A, PEAK1, CNOT6L, ASXL3, WDR1, ACSL6, PDXDC1, TMEM26, DUSP28, GOLGA5, FLOT2, PRKCZ, RTP1, RC3H2, VDR, STX18, BNIP2, CHRM3, FAM185A, RBAK, CACNB1, NUTM2G, BPI, MYO9A, ECE1, FAM129B, PBLD, STX16-NPEPL1, NEDD9, MS4A3, SORD, GAB2, EIF1AD, TMEM132E, IRF5, RFX3, RASAL2, RNF217, CENPP, C9orf170, GUCD1, DIS3L, DNAJA4, HCN4, CST9, LURAP1L, NFS1, PRELID2, NCKAP5L, COL4A3, FOXK2, KIAA1467, ST8SIA3, HIC2, ADCY1, FNDC3B, ALG1, AGGF1, KCNC2, SEPN1, PPP2CA, LGI2, MYO1E, TRIM65, PPP1R12B, RGS7BP, CACNB3, UNC5C, C15orf53, ZNF546, CCDC126, CREM, ANKS6, HNRNPUL2, TP73, ACOT13, RUFY4, DICER1, RAB6B, RBM24, DUSP6, KIF1B, RAP1A, ZBED1, PI4K2B, ERBB2IP, TBC1D16, FBXL19, ZBTB37, TBC1D3G, CACNA1E, ZNF707, LPAR4, ACACB, ELOVL4, TMEM108, OAZ2, TP53BP1, TMEM161B, CD5L, CT62, STOX2, SS18, VPS33B, PSMD9, SLC35G3, ENTPD1, AP2B1, MAX, MCTP1, PAXIP1, AC008394.1, CBFB, PROX2, OPHN1, KCTD21, ITIH6, NFIB, MED20, SULT4A1, VASH1, GRID1, HLA-DQA2, SLC6A15, IRAK1, C1orf213, AGXT2, CASP2, THOC5, CSRNP1, BAG4, ITCH, RP4-695O20__B.10, SNX27, KCNH7, RND2, USP37, EIF5A2, GEMIN2, TEX19, RPS6KA1, PSME4, ZNF80, B9D1, NCOR2, FHAD1, ABL2, ANKRD26, GP6, SP9, SCN4A, LAMTOR3, RFXANK, STC1, GJB7, ANKRD13B, CDH11, WDR63, TEX26, MTMR6, ANK2, FAT4, SLC25A39, ACSM2A, ELOVL6, ZNRF3, AMBRA1, C15orf39, H6PD, MED15, TOX, SORT1, RASA2, DNAH10, CELSR2, FBXO48, LRIG3, MKNK2, KIAA1644, SGPL1, SLC4A4, EDN1, CRCP, TYSND1, PTPN14, TMEM232, ACSM2B, SHISA6, TBC1D25, EHD1, TBC1D3H, GRHL1, RHOBTB2, LANCL3, NCLN, DPF2, FRMPD4, GOPC, PRDM15, CSF2RB, TARS2, HIVEP2, FOXS1, LYZL6, HNRNPA2B1, RNF175, TIMM17B, UBR2, ITGA1, NFAM1, KLHDC4, TMEM135, SP3, BCL2L2, C1orf109, CDK19, SHPRH, PCGF6, CCR7, VANGL1, OTUB2, AC079612.1, CREB1, SYT10, COL27A1, DNAH10OS, PRAMEF18, TMEM116, CACNA1B, ARMCX4, C2CD2L, KIF18B, KIAA2026, FBXO45, FZD4, NUDT16, C22orf46, TTC21A, NKPD1, HOXD9, MTFP1, IAH1, KIAA1598, ZNF787, PROX1, DIDO1, TTC30B, DTWD2, PPP1R37, TGIF2, ATXN1L, SLC38A9, ZNF148, IRF1, FOLR3, CALM2, GCSAM, KIAA0556, ZBTB34, TMEM260, BEND6, C2CD4C, FGFR2, PHKA1, QSOX2, P2RY8, ZNF75D, KDM4B, CDC14B, FCRL5, EPSTI1, C4orf19, DST, KIAA1614, MAP3K9, SNX32, SLC22A13, RASL10B, TCP10, UBR7, TMEM170B, EDEM1, USP46, C8orf33, SNX24, AQPEP, AC145676.2, ATP10A, RAF1, CCNC, PPARA, PNPLA1, STX6, MAP6, RAVER2, ARHGEF9, SH3BP4, CREB3L2, LRTOMT, CCDC71L, DPP9, DDX54, DNAL4, PHOX2B, PDE8A, PRSS35, SP140L, NAA15, UGP2, FAM174B, C3orf62, SOGA1, PHF16, SH3RF2, CPLX4, TMEM231, DNMT1, FAM71F1, RNF144A, FAM53C, RNPEPL1, RP11-766F14.2, C7orf43, PIK3R5, HES2, C1orf210, ASB4, PKHD1, MRAP, SLC39A13, ABCB1, TSPAN12, RP11-1407O15.2, FBXW8, C10orf105, CACNB2, NPHP3-ACAD11, HK2, CASZ1, ABLIM3, SYT2, MAMDC2, LRRC8A, ZSWIM3, ZNF396, RHOQ, GPR157, TINF2, MGA, HYAL4, NTRK3, PAPOLB, KRT15, LMOD1, ASTN2, RAB8B, ZNF304, CYTH2, GAB4, AGPAT3, SNX33, SETD7, TREML2, PLEKHG5, HCAR3, HCN1, TMEM136, METTL21A, CNNM4, LETM1, POU2F1, ARHGAP20, CALCOCO2, RASSF3, KIAA1244, REST, TMEM50A, EPOR, SLC8A2, VCPIP1, LMLN, CNNM1, FBXO42, MGAT5, SCN4B, ATP5G2, PTCH1, CPM, ATAT1, GKAP1, MAPK7, FKBP15, FMR1, NCAM2, PARP14, CAMSAP3, KRTAP5-8, CER1, AGPAT4, CAMK4, LHX9, CHD8, ARHGAP40, NOX5, C12orf74, MAVS, IMPAD1, FGF5, TMED9, EXO5, CDH23, RP11-121M22.1, HIPK2, ABCC11, CNR2, TRIM14, BCL2, LONRF2, FUT1, DUSP7, PSG8, NICN1, FOXQ1, SLC6A17, C1orf110, LACC1, TUB, SNURF, KIRREL, BMPR1B, TBC1D3F, ZCCHC8, TECPR1, THY1, SKIV2L, ZNF254, C17orf103, SLC46A1, PRR23A, DGKH, RBM38, CNOT3, SLC30A2, ZNF687, LLGL2, TAZ, GALNT7, DENND6A, COL24A1, NDST1, SCN2B, HOXD1, ZNF559-ZNF177, IFNG, HNF4G, SCML2, IL22RA1, RIMS3, KCNB1, AGBL4, SERPINB8, ATOH8, CCDC169, CBX7, TPPP, UBOX5, PHF20, SLC2A10, CD69, HIF1AN, ZNF362, PHTF1, ago-02, WIPF2, MTMR3, EPHA8, PTPRB, NWD2, KCNMA1, ATIC, FMO2, GPR160, SGCD, DRP2, C17orf102, TBC1D20, COL21A1, TXNRD1, UBE2L3, CMTR2, CCDC120, C6orf132, ANO3, APOBEC4, KMT2D, SAMD7, TRPV5, EPS8L2, HCAR2, ENTPD7, FAM222B, CYYR1, ATXN1, HIATL1, HRNR, ZNF12, FOXP2, C6orf223, IFNAR2, HDDC3, PANK2, VEZF1, PSME3, MAT2A, KIAA0753, ZNF652, PVRL2, QPRT, HEMK1, GALNT16, ACTR8, AMDHD2, PDE7B, KXD1, ANGPT2, ZNF76, CHRM5, KBTBD13, NT5C3L, NCAPD3, BRCC3, EDC3, CFLAR, COL11A2, ZNF592, SEC22A, GRAMD2, C5orf64, SH2D7, OPALIN, KMO, LRRC25, FANK1, C7orf72, APBB1, VPS37B, SRSF6, LL22NC03-63E9.3, CAPN14, LANCL1, MARCH7, CHRNA2, CDK15, DIEXF, CAB39L, ZDHHC15, FAM178B, ARHGEF3, LIF |
| miR-451 | ATF2, CAV1, PCCB, SPC25, SRP68, OSR1, EREG, TBC1D9B, PSMB8, RNF217, AC114546.1, SNRNP70, CDKN2B, CMTM6, CUX2, CLHC1, CAB39, TSC1, CHAF1A, GRSF1, C11orf30, FBLN5, SAMD4B, ZMAT3, ADAM28, MIF, CTBP1, ITPR2, RBM4B, PTTG1IP, AEBP2, DBX2, SNRNP48, RAB14, PHACTR1, KLF11, KIAA1217 |
| miR-126-3p | PTPN9, PLXNB2, CTD-2162K18.4, KANK2, ITGA6, ZNF556, ZNF219, SPRED1, PLK2, IRS1, SLC37A3, PMM1, CAMSAP1, FBXO33, C20orf26, DIP2C, TNFRSF10B, EFHD2, SLC7A5, ZNF131 |
| miR-126-5p | ESRRG, RFX4, MDM4, SKOR1, HOXA13, A2ML1, HSPB8, AL353698.1, EGFR, MAP3K2, INPP5D, GRIK2, TRPS1, TRIM8, S1PR3, ARL11, FAM168A, SUGP1, GNE, REV3L, CASK, DENND1B, ZBTB7B, FGF7, NCR3LG1, PREX2, NAT1, MFAP4, UBN2, GABRB2, CPED1, MACC1, BAI3, BZW1, SOX5, SH3BGRL2, FXN, NAF1, ZNF738, EGFL6, NFYA, AP3B1, TSC22D4, KIAA1456, ZNF33A, PLCB1, GABRA4, HOXC12, ANO5, CALHM1, KIAA1644, C11orf87, SLC25A53, SULT6B1, L2HGDH, FAM9C, TNFAIP8L3, ING3, TMEM56, TBC1D3C, PDZD8, PAX2, GIN1, WFDC13, RABL3, TBCA, SLFN5, TMEM41B, FAF1, EIF3J, PPP4R4, CHI3L2, YIPF4, BRWD3, LAMTOR3, CAMK2A, GGT6, PTPN20B, HOXC8, RAB30, KL, NDUFS1, EIF2AK2, CSRNP3, JARID2, RASAL2, ROCK1, PTPN20A, JPH1, PTPN12, PLEKHG7, AMFR, PDE7B, COLEC12, MBOAT1, CLEC1A, FBXO6, GCLM, MGAT3, TMEM182, MMRN1, RP11-849H4.2, CD44, LYPLA1, MAN1A1, sep-07, CHD1L, FLT4, STPG2, SCD, ZNF687, CHMP5, TRDN, OSTN, MIER3, MAK, KIAA2018, CCDC88A, ZNF33B, MR1, FLRT2, PDLIM5, IL23R, CHM, INO80D, FOXN3, ZNF627, HOXD3, CNOT4, EIF2A, CCDC13, AGAP7, NPAS2, GULP1, TUBGCP4, CSNK1G3, DDX59, NCOA7, TMEM33, GRID1, LRRC55, TTF2, EYA1, PPIL1, HIPK2, KMT2A, SERF1A, ADAMTS6, AC025278.1, GJC1, SOX6, CD84, ZNF519, NCOA2, COMMD2, IDS, DICER1, AKAP6, PNRC1, STXBP4, RBM26, MAT2B, ZDHHC15, STC1, STEAP2, SKIDA1, PIK3CA, HOMEZ, C8orf49, CCL28, CREB1, C12orf55, COMMD10, FAM27E3, QRSL1, ERGIC2, FZD3, CDCA7, RASEF, FPGT, TMPO, TMEM237, GPA33, C12orf50, CDK13, DNM1L, EREG, CYS1, GPR85, RORA, BICD2, SLC6A15, PPARGC1A, ZNF354C, GPR88, ST8SIA3, RSBN1, TBC1D15, LPP, LRCH2, LTN1, C4orf29, MSRB3, EBF1, TMOD2, HOXB6, HELZ, C18orf25, DTL, RGS18, FAM111A, PTPRD, CA1, AC130352.1, PTPN4, TRPC5, RGR, FBN2, HSD11B1, ZNF207, KRT78, ETV1, SKAP2, CADM2, CSF1, USP12, KLHL32, CCDC179, SPATA2, WDR35, AP1G1, NSUN3, DHX33, SPAST, MAPK10, OTUD4, EFCAB11, ZNF503-AS2, RAD50, ANKRD32, SLC2A13, N4BP2, ADCYAP1, FAM102B, LPPR5, CPEB4, DIRC2, EPHA7, GBP1, UBE2W, GNAO1, KCNT2, ARL13B, SAMSN1, ZBTB7C, FAM199X, LRRC42, GOLGA6L4, VIL1, NUDT12, NWD1, MTL5, RGS5, MANEA, ARSK, SPATA6, RUNX1T1, ZFP30, F9, ZNF10, RAB8B, FBXL8, KIAA1468, HPSE, C2CD4A, TMEM26, HECTD2, DOK6, PI15, CMC1, PAX9, THAP3, RP11-463J10.2, MON2, ZC3H6, EXPH5, TCHH, LANCL3, CLCN3, MICU3, CREB5, TMEM65, NHLRC2, ZC3H12B, ISG20, ACTR1B, KCTD3, C10orf126, FSD1L, SPX, SGPP1, C2orf69, DAB2IP, PDGFD, SELT, ADAMTS4, TNRC6A, SMAD4, CACNB4, CLEC12B, RNF19A, C7orf55-LUC7L2, EVX2, PLEKHH2, ASAH2C, FAM27E2, GRIA2, ZBTB8B, CALHM3, C8orf37, AZIN1, PRRG2, MS4A1, FAM216B, PAX6, BCL11B, AP000708.1, PGM3, ZC3H8, CBFB, BLZF1, CDH8, SYNPO2, SPATA18, FAM27E1, CNTN1, CELF2, ERO1L, DRGX, CTDSPL2, PRTG, CD200R1, MCHR2, SREK1IP1, EFR3A, EYS, PTGS2, ZPLD1, ELK4, TNKS2, C9orf41, PDGFRA, ERAP1, COBLL1, SRD5A3, RBM46, FBXO3, LDB2, IL7, ZFY, PRRG3, ATXN7, ASAH2B, NFAT5, AIMP1, MMS22L, TSHZ3, LOX, TMX3, RP11-67H2.1, MRPL42, IFFO1, STK17B, GPR12, XRN1, ZC3H7B, DMRT2, DDR1, SLC35A5, MBOAT2, NDUFA9, SPICE1, GPBP1, BRAF, SOCS6, VSTM2B, ABCA5, ASAH2, CAMK4, SRFBP1, IL15, LONRF2, GLIPR1, ZIC4, ZNF695, MKLN1, RNF175, ZFHX4, SSX2IP, RALGPS2, RIMS1, REEP3, PAQR3, CORO2A, POLR2K, GXYLT1, BRD3, ATF7IP2, PRRC2C, CEPT1, BACH1, E2F7, SGCB, HMGA2, METTL2A, FAM165A, FAM183B, LHX9, MYEF2, C21orf62, MBLAC2, RBM12B, CD2AP, FRYL, DACH1, TMEM2, SSBP2, RTKN2, RFTN2, TAOK3, CHD9, CYB561D1, UBXN2A, SLC30A1, OCLN, ZNF703, ZNF148, HMGXB4, TET1, SERF1B, AQR, FAM78A, ARID2, DST, FBXL14, PLA2G12A, PPP1R1C, DMXL1, RPS6KA3, AHCTF1, BNC2, CPEB2, PLAG1, KPNA5, POU2F1, CPNE8, C9orf114, ATXN7L1, SYS1-DBNDD2, AGTR2, PELI2, ZNF583, GOLGA8F, REXO1L1, MCTP1, FZD6, MIB1, ZC3H13, GJB2, TFEC, ITGB8, MAP9, ZNF777, PHLPP2, SCRN1, PCSK2, SEMA7A, GPATCH2L, HOXB2, DYNLT3, C6orf89, PPP1R10, BICC1, MLPH, RAPGEF6, CLASP1, TFPI2, LCP2, FPGT-TNNI3K, ITFG1, HOXB5, OXSR1, PRIMPOL, IGJ, GABRA5, STYX, PDE10A, DUSP28, AGPS, PLOD2, SLC25A30, PTAR1, SLIT2, KLF17, EDA2R, KCND2, GPR110, PYGO1, GOLGB1, C12orf40, C5orf47, NF1, UGT2A3, SAMD4A, GTF2A1, SH2D1A, SC5D, CEP97, TNFSF4, NR2F1, ARHGAP20, PPP1R15B, PDE3A, CHRNA5, FAM13B, FAM135A, VAPA, DCLK3, CDK6, ZNF254, GNAQ, SBSPON, KIAA0355, ICK, ELAVL3, USP47, RP11-108K14.4, FAM76B, ERBB2IP, ZIC1, TCEA1, DIMT1, TNFAIP8, ITGA8, BTC, SEC62, CPD, MOB1B, LHCGR, SLC5A3, PCGF5, ROCK2, SRSF12, GUCY1A2, PRKG1, DCUN1D3, NPAS3, FOXP2, CCNT2, CHST7, SEMA3D, ZNF566, TRHDE, NEUROD6, ZNF720, C5orf51, SLC17A8, UAP1, SORD, U2SURP, PTBP3, DYRK2, MPP7, TMEM263, ABCB5, PAX5, PANK3, ENPP5, ZCCHC5, LRRC3B, CCDC149, MOG, HELB, DAPP1, VTI1A, ZNF536, PURB, SEC61A1, GPM6B, BMPR1B, ZDHHC21, ZNF681, AXIN1, FAM198B, ZNF383, G3BP1, IL36G, TRAF6, RBBP9, SNX18, ZFP14, APOPT1, PDZD2, C1QTNF7, LIMCH1, JRKL, RP11-169F17.1, C11orf45, BAG3, EPHB1, GLRA3, VWC2L, POFUT1, SLITRK4, BTF3L4, MYOZ3, HCN4, CCPG1, PTGFR, HRH4, FAM151B, SPRED1, MTCH2, RP2, HTR1F, SLC35A3, EYA4, NPR3, KRAS, GMNC, NEGR1, PLEKHB2, TFPI, MAP3K7, ABCD3, FMO2, FAT3, ADO, PIKFYVE, SMARCC1, NLE1, GSPT1, INSC, FAM63B, PPP1R12B, PAPPA, SOS1, BTAF1, ZNF81, DLK1, ZFR, ARL5A, KIRREL, MED14, TNFAIP2, QPRT, TCFL5, GIMAP8, FAM129A, HMGB1, TMEM248, NR1D2, PFN2, SLC2A11, GMPPB, PNLIPRP3, SLC35G1, SYT5, POU5F1B, APPBP2, RUNX2, RFX3, CBWD3, ANGPTL7, DCUN1D1, SPRY3, POC1B-GALNT4, NDUFV2, TCF12, NEUROG2, VWA8, OAS1, ELAVL4, UBTD2, ATF7IP, MSR1, GAB1, C1GALT1, GOSR1, RBM19, C1orf141, SRRM1, ANKIB1, CCR1, CEP78, LRRC8B, ZDHHC17, NRK, RCOR3, IKZF2, ENOX2, PDE4DIP, PTEN, C21orf91, MARCKS, C12orf65, KLF12, IMPAD1, CBLB, HGF, HEATR5B, CYP4F3, HLF, VTA1, ABCE1, ASXL2, USP10, IGSF1, CDC14B, SEPSECS, ADK, TMEFF2, BTBD3, GPR27, GABRA2, SPAG9, MTO1, SOD2, SLC26A4, MITF, TDRD3, PAWR, ERICH4, ANKRD34B, LMO7, ENPEP, MCTP2, FAM189A1, PRUNE2, APOLD1, EZH2, SLC12A2, MLLT10, OSBPL8, SLC41A2, ARMCX3, TRAPPC13, MDGA2, C7orf57, HIBCH, ZMYM6, TOB1, ARHGAP28, HDAC2, BTLA, ANTXR2, PAPD5, PRKX, NETO2, C4orf46, RAPGEF2, SREK1, FMN2, PROCR, ENPP6, FAM221A, CUL2, UBXN7, RASA2, TMEM135, FAM179B, RINL, SRSF10, ERMN, SETBP1, GRB2, PEX5L, PHTF2, NIPA2, ATL3, RCOR1, ASPN, JMJD1C, TSHR, ZDBF2, SHISA3, TWF1, PIP4K2A, TRDMT1, GABRA1, ZBTB38, MOB3B, STARD4, SGTB, CBWD6, TENC1, CCP110, LMBRD2, KCNJ16, ARHGAP18, CA3, ABRA, ZNF280D, GATA3, SLC7A2, TTC39C, RP11-73M18.2, NBEAL1, KIRREL2, PNPLA8, HDAC4, SH2D4B, ADAM22, BEND3, CASP3, BCAT1, MYO3B, KCNJ3, ABCC9, SPAG17, STRN3, SYT14, TTC7B, ALCAM, KIAA1033, VCPIP1, HLTF, RNF217, STAM2, EPGN, ERVFRD-1, KIF15, ZBTB1, MED17, CCDC38, FAS, ZNF624, CHRNA7, RBM41, SLC38A6, LACC1, NTRK3, FGGY, ATG10, MT1H, DDIAS, AP1S3, ZBTB21, ZNF674, ABCA1, PIK3C3, ATXN1L, TRIM64B, GABPB1, FAM8A1, PALLD, HBS1L, USP15, P2RY12, GCC2, MBD5, CHP2, PDS5B, DCX, C10orf53, HACE1, VPS13C, CRISPLD1, SNCA, SLC30A6, USP14, SLC26A2, WDR48, AC008079.9, C5orf24, DBNDD2, REG3G, ONECUT2, KLHDC10, NREP, SLC10A7, CHPT1, LATS1, CLLU1, SPACA1, MID1, CSMD1, SLC16A7, MYBL1, TMEM39A, RAD21-AS1, VGLL1, NEUROD4, NCALD, KCNV1, C8orf34, MAPK1IP1L, PHLPP1, SCAP, TMED7, ZBTB26, AK9, USP46, ATP6V1G2, METTL6, TBR1, GOLGA8G, MAFB, PLCXD3, GLS, CUBN, TRMT10A, BMP3, PCDHAC1, RB1CC1, RP11-766F14.2, CPA3, E2F6, PRDM8, TOX, SEC11C, SHROOM3, ZNF546, ATF6, NDFIP1, NUAK1, UHRF1BP1L, TCHHL1, KCNJ2, DSG4, DLGAP1, RP6-24A23.6, WEE1, C15orf41, SLC6A5, MMP16, KIFAP3, GTF2F1, C8orf4, LONRF3, TET2, DGKB, DNAH5, SMC5, RAD21, LRRTM3, KBTBD6, PRKCA, FAM3C, PAK2, CNTNAP3, ADAM9, RAB9A, ZNF385D, GNGT1, ZBTB2, NFIA, OPA1, IL17A, NCAPG2, PRKAA2, C18orf32, PMS1, TMED2, DKK2, FYB, ATXN3, CALD1, BICD1, UACA, AL137003.1, ZNF347, ACSM6, NCAM2, NAA50, SFRP4, ANGPTL3, MTDH, EFCAB7, PTPRB, NLGN4X, DHFR, MRS2, CHCHD3, NR3C1, HIPK3, SOX13, CA12, CLOCK, PPM1B, TBC1D20, GSKIP, CCDC68, TMTC1, TMEM27, CHL1, CHRM2, KIAA1024, LINGO1, ZZZ3, ISPD, UBR1, CTAGE5, GLE1, ANGPT1, PEG10, CLIC2, DVL3, XIRP2, CRY1, SNX10, KBTBD7, KCNG3, SDE2, USP9X, ASPHD2, FAM201A, ITM2B, OTOGL, GCNT3, ZNF793, AL359878.1, PRAMEF13, MBD2, ITSN2, ADAMDEC1, LMLN, RALGAPA1, CENPJ, SESTD1, EXOC5, PLP2, GDF6, AMACR, HHIP, ZNF492, SNX15, SLC24A4, ATMIN, PLSCR4, BBS12, PGM2, ZNF385B, LRRC57, C5orf30, BPI, PNN, TMEM257, SLC25A37, C22orf45, DNAJB4, DLAT, HECW2, PTER, PGC, C4orf26, REEP5, IGF1R, SLC26A7, ZAK, TGFBR1, TGDS, RBBP6, UBE2H, ZFPM2, DLL4, ZNF273, NUPL1, ERCC6, SPATS2L, WDHD1, CCDC53, FAM160B1, GOLGA8IP, FGD4, EIF3H, CCSER2, AKNA, SLC35F5, HNRNPLL, CCDC50, TMEM123, CHAMP1, NABP1, DMBX1, KPNA4, ZNF567, MBL2, ASPH, NACC2, B3GNT5, NSUN7, TMED5, GINS1, PHYHIPL, CTD-2054N24.2, COL11A1, CTAGE9, FBXO36, FRZB, TEX15, NUS1, SYT10, KATNAL1, POLK, FOXR2, SSPN, FAM47E-STBD1, STRN, ZNF615, CASP9, CRB1, TLK1, EP400NL, TBC1D19, IRX3, EIF4A2, SLC25A24, JKAMP, LRP6, ANXA7, TCF24, CDH19, FNTA, PPM1A, EVI2A, C1RL, NCKAP5, LCLAT1, CMTR2, FAM84A, C1orf27, ARHGAP24, UTY, CEP170, LYRM5, SLC38A4, GALNT4, YTHDC1, PIGK, MAP4, SGIP1, TBCK, GOLIM4, PATE1, PPP6R3, MTAP, EARS2, PSMA5, PARP12, SEMA6D, SASS6, KIF3A, CLEC1B, SPDYE3, AC005754.1, VMA21, ZNF333, CTCFL, TRNAU1AP, CTBS, PPIL6, COL12A1, HMGCS1, PANX1, MRPL57, SLC25A40, TRIM33, IGSF10, HIF1A, RSRC2, CMYA5, AP1S2, FAM196A, RAB11FIP2, AK4, ARPC3, BCL2L2, ACYP2, SECISBP2L, PUS7, TRAM1, SESN3, SLC7A4, TMEM50A, VGLL3, FGL2, DCDC2, SLC1A1, PCDH18, KIAA1671, ATR, ST6GALNAC5, CCNYL1, MYSM1, KAT2B, THEMIS, CXXC4, IMPG1, ITCH, CCDC141, MBNL3, TOMM34, CRLS1, NOVA1, ARL4C, SORBS2, TMEM70, EBAG9, GRIN2A, HNMT, GAS2L3, HOMER1, RGS4, RAB9B, PRAMEF14, NFIB, ZBTB10, C15orf27, ADAM8, DUXA, FGFRL1, BMP5, KCNQ5, SIX4, SLC16A9, MLK4, FRG2C, FNIP1, OTUD3, ANTXR1, PARP8, UNC79, PMP2, UTP14C, MAP10, SCN3A, SLC30A4, NANP, CLDN12, TRAF3, HIST1H2BF, AC093510.2, BPY2, LGALSL, MS4A3, BCAP29, HSPA4L, MEF2D, LRRK2, PPP4R2, AHR, SHH, IL1RAP, ZNF208, ROBO1, PIK3C2A, C7orf63, AKR1B10, RRP15, GFPT1, VPS13A, LRRC9, NCOA6, MED13L, KCNB2, PMPCB, AKAP4, PKIA, EBPL, NTF4, INPP4B, KERA, FAM122B, PEX3, C6orf118, SLC22A24, HS3ST3B1, ZNF100, FBN1, ERLIN1, PARP11, RNF41, TMTC3, LRRC8D, KRR1, RHOBTB3, RXFP1, KIAA1549, PRR23C, CCDC59, UBE2G1, BAIAP2L1, CSF2RA, PRDM1, ZXDA, BEND4, RAB3IP, HECA, NXT1, OR51E2, PM20D2, DCP2, MCM3AP, EPHA5, CDK19, NAA30, SUMF1, ADAMTS5, ELF5, YES1, RAB2A, FGG, RCBTB1, GPR180, MIER1, C14orf23, GLUD1, ABCA10, GRAMD1B, BHLHE41, EPT1, ZNF461, SLC1A2, MAP7, GRM1, CCDC132, RAB3GAP2, DCLK2, ZADH2, RALGPS1, ZIC2, DLG1, GABRP, CUL3, GSK3B, DDX10, RPRD1A, GREM1, OSBPL11, UPP2, ULK2, NOG, SPO11, ST13, GPR137B, PPAT, EPHA3, ZFX, ZNF23, FABP4, SPOPL, NHSL1, GALNT5, MGP, NCAN, FSHR, HP1BP3, ANGEL2, BCLAF1, XIAP, HINT3, GPR64, USP45, CRCP, GPR151, SOCS3, TOR1AIP2, AP000783.1, AMMECR1L, DCAF4L1, SNX13, GBP3, SLMAP, B4GALT4, HPS5, ALDH6A1, SPIN4, SOCS4, ATG12, IGF1, FBRSL1, LCORL, GPBP1L1, FAM120A, SMIM14, MAGEE2, MEGF10, RP11-1220K2.2, FGFR2, KATNBL1, GRIA4, UGT8, CLDN22, SDPR, WBP2NL, GNRHR, CNTN4, HEATR1, ITGB6, IREB2, RNF103, EMCN, TOP1, ITGB1BP1, ARMCX2, USP51, RIMBP2, TERF2, RP11-152F13.5, NFXL1, SLC35E3, CRK, WDR17, ARNT, CREBBP, UBE3D, PDE1C, CHEK1, DCK, TMEM183A, SPDYE1, UPRT, PPP1CB, PHKA1, GBP7, SYAP1, BARD1, B2M, UGT2B17, UBE2V2, SP1, ATG3, RABGEF1, MUC22, BNIP2, RMDN1, CDH9, LINC00493, FAM172A, YWHAZ, TMEM236, CDK12, ZNF800, FURIN, STK3, BCAR3, DSC2, TROVE2, TDG, CNPY1, REPS2, TCP10L2, ZBTB6, HNRNPA1, DPPA4, MSANTD4, CASR, PRKACB, ZWILCH, LIMS1, PIGX, SLC4A7, CANX, TSC22D2, FIGN, C15orf61, C14orf37, EEA1, MAGEB5, ATAD2, EGLN1, TTLL7, ASB4, ZRANB2, YAF2, GLRA2, PLA2R1, TVP23C, KLHL24, FAM23B, ZNF697, ULBP1, COL4A4, XKR6, PRAMEF1, TTC37, LDLRAD2, PHF23, NEUROD1, IYD, ATXN3L, PCNX, RAG1, HERC4, BTN3A3, C15orf53, TOX3, RARRES1, TMEM55A, ZNF776, RASSF6, ZNF770, KLHL4, SNCAIP, AP1AR, GMCL1, ZNF26, COX5B, SDC2, PTPLAD2, OSTF1, CHRAC1, FNDC3B, IL1F10, MXRA5, ZNF253, KDM6A, MYT1, OSMR, STOX2, ASTN1, PCDHB13, CRTAM, THSD7B, LUZP2, FAM46A, AL953854.2, HPGD, PSG1, C14orf64, YAP1, SNRPF, ERI1, TMCC1 |
| miR-145-5p | TRIM2, SRGAP1, CACNA1D, CAMSAP2, SEMA3A, RBPMS2, DERA, RTKN, HNRNPH2, MDFIC, YES1, GRB10, TBPL1, LRP5, DOK6, CTNNBIP1, TEX37, ABHD17C, CLEC16A, PPP3CA, SKP1, ZNF788, SNW1, SCN2A, ACTB, ERLIN1, RBM20, CYP46A1, PCBP2, ADRB3, NOTO, LRCH2, TBC1D14, PHKB, CCDC39, EBF1, BLM, OS9, RAD23B, ZFP90, SCFD1, EXOC8, GOLGB1, TFAP2A, ST6GALNAC3, CMAS, EMX2, FLNB, HS3ST4, BRWD3, RGS7, PLCL2, ZNF704, RBKS, FOXE1, LPXN, EPB41L5, SENP2, PTGR2, PRKD3, ABCE1, NLN, MMP16, NAIP, ADH6, AGPAT3, EPT1, LCOR, MAGI2, RP11-212D19.4, CYFIP2, SIRT5, MARVELD2, LRRC16A, SAMD12, ZDHHC9, ZNF525, RBPMS, NWD1, TMEM260, HIC2, ZNF570, CAMK1D, KCNJ12, C7orf55, FAXC, CAMK2D, PIEZO2, SMCHD1, ABCA13, IKZF5, BZW1, SMAD3, RANBP2, WWOX, SMAD5, UBA52, ARHGEF40, AC092329.1, CDC37L1, UBA6, UMODL1, ENC1, ANGPT2, C1orf56, PALM2, MYLK4, DPAGT1, UBR7, TTC26, ACBD5, SRSF6, ARHGAP6, AMOTL2, ADCY4, CCDC57, MPZL1, AKIRIN1, EIF3G, PCDH20, ARMCX4, TAOK1, CEP350, SLC25A31, GABARAPL1, SLC38A2, NET1, FAN1, TMEM222, SMIM18, SESN3, SLC52A1, HMGA1, SMLR1, ENSA, ATP8B4, LHFPL2, WDR61, RP11-166N6.3, SIK2, TMEM206, SMARCD3, TRPV4, PDE8A, NEDD4L, PPP1R9A, CTTNBP2, TLE4, SORT1, TTC9, RPS6KA4, FAM193B, HSPA14, SLC1A4, ELF5, GDPD5, MIPOL1, ZNF107, TMEM167A, BCL6, MEIS1, TPP2, PRLR, POTEM, ZNF611, LHX4, COL20A1, PSMC5, PODXL, ZNF131, LCP1, APH1A, XPO1, DBX2, MRPL48, CCDC176, STARD8, INTS8, NFIL3, KIAA1958, FUNDC2, USP38, CLINT1, DNAL1, DLX3, ZNF720, CCDC30, C19orf82, MAPK4, CABP1, THRAP3, DNAH3, ZNF846, PTPRK, ATP11A, FBXO43, DENND5B, RGL1, CMAHP, WDR17, COL6A6, GABARAPL2, DYNC2LI1, NAA25, UBE2Q1, MBL2, CACHD1, GCA, RAB27A, EMILIN3, RASSF5, EXT1, RILPL1, TBC1D15, MIR3654, ZNF441, PAPD5, MBTD1, UGCG, RYR2, ASPHD1, COX16, MAP3K1, UQCRB, GLMN, CLCN3, DDX49, KCTD15, DAO, HMCN1, ZNF282, KIAA1598, SACM1L, ABCA7, F11, JAM2, KCNS2, NPHP3-ACAD11, ZNF493, SYT14, SMIM19, ZNF730, SYPL2, ZNF440, FAHD1, ACACA, CPSF3L, DLX6, RPE, ALMS1, ZBTB44, TJP1, BTN2A2, CACNA1C, TXNDC9, HPRT1, ZNF91, AGPAT4, MED13L, IQCH, RBM6, GALNTL5, SLC7A2, ACBD3, RBBP5, CDCP1, GPHN, CIITA, SERAC1, VPS54, HSPA5, NR4A3, ZNF776, CDH23, CAPS2, MST1, EML4, DLEU7, ZNF732, DCDC1, IL33, BRMS1, PDGFD, CELF2, SLAMF1, FAM203B, CCNL1, SFXN1, SLC25A25, STXBP4, TMEM117, LUZP2, ZXDB |
| miR-23a | ZNF138, ZNF225, SESN3, AC092329.1, PDE7A, KIAA1467, MRC1, NACC2, PRLR, CELF2, ZBTB44, TSG101, ZNF273, MRC1L1, GOLGA6L4, PPARGC1A, FBXO32, ATP6V1E1, CXCL12, ZBTB34, SEC24A, AMBRA1, SOCS6, PDE4B, CNOT6L, TOP1, MAML2, CTCF, UBE2D1, SEMA6D, TNRC6A, KLF3, ZIC4, KPNA4, NFIB, RPRD2, SFT2D1, TACR3, SLC6A14, EBF3, GLYR1, QSER1, MAGI3, ZNF559, TMPO, NUP50, MAP4, ZNF676, ANKHD1, SATB1, ZNF720, RALYL, HDX, ZNF669, MYCT1, ZNF655, CAB39, ARHGAP20, ERBB4, CELF1, KIAA1524, NDFIP2, ZNF493, RNF38, POU2F1, PKP4, HOXD10, TRAPPC6B, POU4F2, ZNF721, TMOD2, DPY19L4, ZNF233, MCFD2, RP5-1052I5.2, COG3, TNRC6B, MAP3K1, LYPLA1, RP11-595B24.2, TJP1, NAP1L5, GSTM2, NUTM2G, MET, GABRG1, LIN54, JMJD1C, VCAN, NLGN4X, SYT4, PPP2R5E, ZNF525, TNKS2, DLG2, GPR64, USP51, DOK6, DEPDC1, MDFIC, ZNF107, AL645730.2, CSNK1G3, ATXN3, POU6F2, ZNF708, TXLNG, VWDE, AAGAB, PRTG, SLC1A1, NUDT21, RAP2B, ZNF223, PRDM2, ZNF226, GPRC5B, TMEM38B, PPP4R4, SHPRH, APAF1, CLCN3, INTU, IGSF8, LRAT, PNRC2, ZBTB43, VKORC1L1, ZNF286B, ZNF267, C3orf52, TGIF1, CRISPLD1, ZFP62, MYH1, MAB21L2, FMR1, COL4A4, MAP4K4, SPRY2, TOX, ZNF208, FRMD5, PATE4, PHF14, AUTS2, RP11-766F14.2, SEC23IP, RAB8B, PPP1CB, ZNF765, PURB, RAB39B, TRIB1, XIAP, KDM6A, PIK3R3, NUFIP2, ZNF91, SATB2, ROBO1, ADAM19, ZNF667, DACH1, NCOA6, HNF4G, STK32A, SPDYE1, NLGN4Y, ZNF292, CDC40, NLGN1, NEK6, ZC3H12C, CHST7, ZNF100, NEDD4L, ZNF730, KMT2C, ZFHX4, ZNF257, MYH4, MUC19, DNM3, SLC35G3, ZNF654, ROBO2, BRWD1, PCMTD2, GNPDA1, ZNF254, ZNF253, EPS15, TXNRD1, KIAA1432, C16orf72, NCOA1, PPM1A, ZNF286A, ASXL3, SETD2, AUH, LPP, XPO1, C2orf69, CSRNP2, CALCR, KCNK3, ZNF28, IL12B, PRKAR1A, MCM9, ZNF701, VPS37D, NTS, SETD8, UBE2D3, TNFAIP3, TOX3, PLAU, MARCKSL1, NPR3, AGAP1, SNX27, GOLPH3L, TNRC6C, ZNF839, PTP4A2, TTC7B, OTUD4, TBC1D24, PPIF, PTGER4, SIX4, BAZ2B, ENDOV, STX12, ZNF480, IPMK, BPY2, SOX11, SLC39A10, IL6R, POM121C, PRDM10, SMS, SPOCK1, CIPC, CUL3, TXNL1, CLEC12B, ATP11C, TADA1, SEC23A, CPEB4, PTPRK, CXorf22, TMED5, MED12L, KPNA5, MEIS2, CNOT4, ZNF43, SNRK, LRRC9, ZNF675, SMEK2, ZNF782, GAB1, SMN1, AKIRIN1, GLCE, GXYLT1, PPFIA2, UBN2, SNRPC, TBC1D15, PDPK1, PRR13, GRM5, ARFIP1, ZNF681, GPBP1, GBP3, MBOAT2, OSBPL8, SIPA1L1, FUT4, ZNF506, TGFBR2, STK4, EXOC6B, FYB, DUSP5, NCOA2, ANKRD50, RAD51AP1, ASAH2C, FAM114A2, SPATA6, FGD4, ZNF805, CPEB2, SLC17A6, AMER1, STT3B, ENC1, CARD8, GK, SLC7A1, MSMO1, CA2, REPS2, SEC14L1, PIK3CB, FAM120AOS, XKR3, FOXK1, ZNF468, NDUFA5, RAP1A, TRIM37, UBE2O, PNMA2, ADCY1, WNK3, CFL2, UBE2R2, ERO1LB, ASAH2B, SLC4A4, TMOD1, CNN2, ZNF716, PET112, BNIP2, ESRRG, FNBP1L, RDH10, RP11-455G16.1, ZNF430, OTOGL, NTRK3, COL4A5, MFHAS1, ZC4H2, AEBP2, PLCXD3, DDAH1, KLRF1, RP11-210M15.2, INO80C, DLX1, ICK, PRR14L, ATRN, ADRBK1, ARID3B, MAGEE1, DCP2, NRXN1, NFIA, CEP350, CNTLN, MTERFD2, SPSB4, DCDC2, MBNL1, ATXN7L3, ADAMDEC1, ZFHX3, TMOD3, NDUFA3, CCDC82, FBN2, DNAJC12, ZNF230, TENM4, RUFY2, ZNF572, UGT3A2, BLOC1S5, ABCC5, HMGB2, DIP2C, C5orf15, RPL31, KSR1, TENM1, GCC2, ODF2L, MTSS1, IPO8, CCK, TGFA, TMEM170A, NOL4, ASAH2, ZNF284, CBFA2T3, DLEU2L, PRKRIR, LIPH, TMEM215, CCDC138, DCLK1, NEFH, APOO, NDUFAF6, SUCO, DNAJC6, ZNF471, ZNF714, MLK4, ENTPD5, HSPA12A, MICU3, ZNF184, VAPA, TBCA, ERC2, BCKDHB, SMN2, UTP23, RORA, DHX15, TENM3, KLHL42, PDE8A, FZD3, ZNF92, TC2N, EML4, NAA15, EMR2, TOPBP1, HELZ2, GAP43, SSBP2, LHX4, LRP5, MAPRE1, ZNF420, SH3BGR, CCDC73, AGGF1, CCNL1, PIGX, MRPL35, STK35, SLC44A5, ARHGEF7, RBPMS2, ZNF431, sep-07, RC3H2, SMAD5, TNFAIP6, KIF1B, CPSF6, ZDHHC17, SESN2, GPR180, ALG6, SMURF2, MYEF2, TEKT1, C10orf68, NEK7, LMNB1, FAM117B, ZNF780B, LRPPRC, ZNF234, BPY2C, RP11-152F13.5, BPY2B, FBXO33, PITPNA, THUMPD3, SCN2A, LPAR1, ZHX1, CAPRIN1, PGBD2, C5orf42, GUCY1B3, CAMSAP2, NUDT11, MACROD2, TLK1, STAM2, RAI14, PRMT9, NEGR1, UFM1, ZNF395, TMED7, CBLB, VCPIP1, PPP1R12A, ARHGAP5, ZNF30, FRG1B, TEAD1, UBL3, FNTA, CGGBP1, B3GNT1, B4GALT4, ATP6V1C1, MATN1, CSNK2A2, IRF1, ATG12, COLQ, MSL2, TRIM63, HOXA3, ATG2B, AFF4, NF1, PKIA, PKNOX1, PTAR1, SSH2, PSMD12, RBM25, C5orf56, MBLAC2, ZNF529, KITLG, CHST11, IQGAP2, SLC30A1, GALNT12, ZDBF2, EXOC1, TGFBR3, SLC7A11, ZC3H13, MAGEB10, URI1, C9orf170, RP2, INTS6, TRANK1, UBR3, RGPD2, ADNP, G3BP2, SIRPA, TMEM263, UBE2G1, MAPK14, PTPN4, OR51E1, LIFR, SPHKAP, MEF2A, ALMS1, USP6NL, PALLD, CD3G, PROSER1, ORMDL1, TAC1, ANKFY1, RIMKLA, MARCH5, XPA, LONRF2, STXBP6, TBR1, FREM1, MAP2K4, CMAHP, CLDN12, PTPRR, NUAK1, PPAT, KDM7A, DZIP3, CBX5, HHIP, ST6GALNAC3, ZNF709, PIP4K2B, ZNF816, ZEB1, CEP85L, VPS53, DDX3Y, RPA3OS, ZNF423, IL21R, RAB11FIP2, GPR155, NCBP1, FAM175B, SNTG1, PTPRB, CASK, RFX3, N4BP1, FCHO2, DCUN1D4, OXCT1, CCNG1, CALCRL, LCOR, PARP11, GPR110, NIN, CTDSPL, PIK3C2A, FNDC3A, FAS, IDH1, USO1, RCHY1, EPGN, UBAP2, TCF24, SNX5, PBRM1, MYO5C, ZNF507, SLC1A3, MAP4K3, ZNF732, GAPVD1, NUCKS1, GOPC, DTWD2, HMGXB4, DENND1B, WDR65, GALNT1, BLCAP, PCDH18, CCNT2, BORA, BTN3A3, MARK1, PCDH17, ONECUT2, HMGN2, RSBN1, ACSBG1, TNPO1, CTC-534A2.2, FCRL3, ZNF697, ZNF677, ZNF670, ZNF737, NNT, SLC9A6, VPS35, ADAMTS6, TAF1D, YWHAG, BICD1, MYH2, CLK4, PJA1, KCNIP4, TFAM, PRSS35, PJA2, AFF1, ANO4, EPT1, MAGEA8, ATP2B2, PROS1, ZNF695, MYNN, IPCEF1, PRRC2B, ZNF682, VTI1A, N4BP2L2, SLC25A36, CLIP4, TRDN, ZNF462, NKAIN2, SNX19, IKZF2, PLXNC1, AKAP11, PRKCA, ASF1A, ATP8B4, SV2B, CSN3, GIMAP7, CAPN6, NKX3-2, NRIP1, ZNF555, NRG3, TOP2B, MADD, EGLN2, AC012318.1, PPP5D1, ZNF287, GLO1, MAP7, CEP78, ZMYM2, GOLGA8B, WSCD1, KLF5, USP53, ZNF492, CCDC71L, ELF2, SLC25A53, ZAR1L, KCNAB1, TBC1D9, HOOK2, PTEN, MARC2, FNIP2, ENPP5, UHMK1, EIF2A, DGKE, BICD2, CREBBP, ZIC1, SRGAP3, MTPAP, LY75, SLC5A3, TMEM71, PABPC5, KLF10, RAB3C, PDXDC1, LGR4, SBNO1, CCDC109B, CCDC125, MEFV, MARCKS, TMED10, ABI1, ESRP1, IQSEC1, RNF152, NAA50, TMA16, ZAK, TSHZ3, TAOK3, PCDP1, ACSS3, TBX5, PAXBP1, PRRG4, RP11-729L2.2, SYT17, HELZ, GMFB, SMIM3, KBTBD8, SGPP1, ZNF429, B4GALT5, MEIS1, POLR3A, ZNF235, FLVCR1, AKAP12, ADRA2A, ZNF280C, MAP3K5, MFAP3L, NUS1, CHSY3, LHFPL2, ZBTB1, ELMSAN1, ZNF579, ANKRD34C, ARRDC3, DHRS11, ERO1L, SLC38A9, USP38, SHFM1, CERS5, FBXO4, SMAD4, MAPK10, HMGCS1, ZNF280D, HNRNPDL, OTUB1, NDUFB8, CCDC158, SMCO4, MLLT4, FRAT2, STRIP2, PPFIBP1, CAB39L, PRDM16, ADH5, KDM4A, ZNF451, HEXIM1, SIX1, CRLF3, TAB3, ZNF569, PPP1R3C, SLC9A1, ZYG11B, SPTSSB, ANKRD27, DIRAS2, ANO5, MTL5, CCDC132, HIVEP2, NCAM2, CCSAP, PTGER3, CTNNBIP1, WDR37, TMEM245, PEX11A, CSE1L, ZNF224, HAUS6, STX17, GPR89A, BMP2K, STXBP5L, ZBTB11, HMGN5, SERTAD4, MMAA, SPOPL, ZFP42, TSPYL4, TRPS1, ZNF189, SCG5, C18orf8, LBR, CRK, SWT1, FHL5, IPO5, EPHA3, ARG1, KIAA1958, ELOVL2, SERINC1, LRGUK, RPP30, CCDC6, ATP13A4, GZF1, TAF3, RCBTB1, PDHA1, MTUS1, ABCA5, TMSB15B, CHUK, ITSN2, LRRC4, ADAM12, ZNF343, HIPK3, PTCHD1, ZNF24, FGF2, NDUFA2, PNMAL1, PRRG1, STAT5B, PPWD1, STEAP2, ZNF136, CEP63, FAM217A, RNF111, MCM3AP, PANK3, LRRC8C, TWISTNB, BTAF1, MIER3, RUNX2, PSD3, HDHD1, GOLGA6L9, ZNF117, PNRC1, DPH6, CTAGE5, DDX3X, FZD5, GNG2, TSNAX, HNRNPU, EYS, IQCJ, ATP8A1, VGLL3, MYL12B, MRC2, PYURF, TTC38, PMP2, CNOT8, GFRA1, INHBE, INO80D, TMEM154, SDHD, WIBG, NPR3, GRPEL2, RNF168, ZNF23, SIM1, ADD3, JAZF1, S100PBP, EYA1, SMC5, PCSK5, HOXC9, MSH6, AL138831.1, REEP5, PLEKHA2, ELOVL5, ZNF717, METTL10, CD93, PNISR, TTLL7, MLIP, SLC7A6, PTBP3, USP28, XPNPEP3, RCN1, RPGRIP1L, ARNT, PLAGL1, NUB1, PPP6C, HMGN3, ANKRD29, PRPH2, KPNB1, EPSTI1, ZNF585A, BLM, TMEM70, ELF4, LASP1, TMEM182, GPR135, ECHDC1, RGS8, TRIM14, MRPS35, NEDD4, SECISBP2, MOB1B, GPR12, ITGAM, ERCC8, RTF1, KLHL12, IMPAD1, ETF1, TPRG1, ENPP4, YOD1, HPGD, KAL1, ZNF461, SLCO4C1, ARRDC4, EIF5A2, MSANTD3-TMEFF1, ZNF724P, SMPX, COL4A3BP, METTL21B, MMGT1, NUAK2, ZNRF2, HAS2, PXDN, SYNE1, ZNF112, TMEM178B, C1orf158, SEH1L, PITPNC1, ATP2C1, NWD2, CHST10, TMED8, PRDX3, OSBPL3, WDR33, PDK4, TWF1, SAMD12, MAF, ZNF845, FOXJ3, SLC26A7, MAML1, TRPM1, ACAP2, ZNF594, PKP2, C8orf59, IYD, HNRNPF, RACGAP1, RP11-1396O13.13, PPP1R13B, C5orf63, MS4A3, CACUL1, REEP1, KLF12, SLC12A1, IL11, RSPH3, RPS27, STXBP4, MCM4, MDM2, ZNF793, STK38L, FGF7, THOC2, PHLDB2, CIRH1A, YES1, GOLGA8F, E2F8, TMEM87B, ALG13, SRP72, N4BP2, OXSR1, RELN, GTF3C4, POU5F1B, NRXN3, HS6ST2, GABRB3, NRGN, SEMA5A, MLF1, ZNF330, MAT2A, LIN7A, JARID2, CDK14, NOVA2, MBL2, PNN, ATG4C, COL11A2, ARNT2, B4GALT6, THSD7B, C7orf60, NUP160, ATOX1, PGAP1, ARMC1, SKA1, ZIC2, PLEKHF2, ABHD6, ANP32B, ZNF664, PTCH1, MDFI, SLC16A6, CHST3, CLEC7A, PCNP, DLGAP2, AZI2, WDR36, ALS2CR11, OPRM1, ATP11B, LPGAT1, MAP7D2, STOX2, ZNF98, FEM1C, ZPLD1, PHLDA1, ATXN7, CTTNBP2NL, USP24, VGLL2, ZNF114, SSR1, VPS26A, CDH17, ANKRD20A3, FSD1L, GHITM, FOSB, OSER1, GPR98, C3orf55, ACSL6, NLK, BTLA, FRA10AC1, ZNF528, TFPI2, GFPT1, GSK3B, SLC17A5, WASL, C15orf40, APOLD1, SLC8A3, OLA1, UBBP4, UNC13A, ST3GAL6, TMEM117, SPI1, COL10A1, WWP1, MAGEB18, ACTN2, ZNF83, ZNF45, CHRNA3, GPRASP1, NRP2, IRGQ, NAP1L3, RXRG, TIMMDC1, KIAA1462, ELAVL2, CPD, SLC25A24, LDHB, RNF217, NHLRC2, AKR1C2, USP45, KLHL24, YWHAE, NR6A1, PLA2R1, LONRF3, ASAP1, TMEM126A, FOXN2, TRIM24, VAC14, BTN3A1, SMARCC1, SLC8A1, IDS, REV3L, FOXL2, HIPK1, AMOT, KCTD12, GCNT2, ZNF227, SENP6, MAS1, HOXC4, F2R, PDE1C, RAB2A, ADAT1, TMEM33, RNF19A, WHAMM, VSNL1, ZBTB26, DCUN1D5, SLC25A4, PRPF40A, LANCL3, STRN, TBC1D4, ADAMTS9, APBB2, C10orf32, PEX5L, AC130352.1, EIF3F, PAK6, ARHGEF6, SNRPE, GAS2L3, PROSC, PFKFB4, SLC35G1, FAM134A, ADAMTS5, PUS7L, ERICH1, GPC4, SNX6, CAMK4, PLEKHS1, ANKRD18B, DSG3, RIT1, FUCA1, EIF2B4, MSRB3, CREBZF, S100A7A, CUX1, DTNA, TSPAN12, SFXN5, GOLGA8A, ELK3, SRPK1, H3F3B, STRBP, CLEC1A, AC135178.1, USP9X, PDIK1L, TREM1, SLCO3A1, BCL11B, PRAME, TM2D1, NDUFB2, HOXC11, CACNB2, EPHA7, KRTAP1-1, ENO2, OPCML, ZNF652, ZFP2, CASC1, C5orf20, A2ML1, ZNF326, CAPZA2, TACR2, HDAC2, RCOR1, MBD5, REST, ZNF596, UGT3A1, PPP2R3A, IL7, AQP9, PAPOLB, C3orf38, C15orf57, ZNF813, C6ORF174, ALG9, RRAS2, GRAP2, METTL3, SIK2, FAM169A, EDNRB, MED12, SHROOM2, LHX9, HDDC2, TEC, ARHGAP27, GLS, CYP51A1, C4orf33, LRRK2, ZNF75D, SF3B3, NOL4L, KIAA1109, RBM46, GPR22, ATXN1, GJA1, MSRA, DCUN1D1, MAGEA1, RAB6B, MLLT3, TMED7, ZNF469, MRS2, AHI1, ARHGAP36, ISCA2, SLC30A4, PAX5, ZNF155, BCL2, ART4, RRP15, ELOVL3, C11orf30, ZNF616, SMIM9, RASA2, FRYL, JAM3, DGKI, LY6K, RYBP, SLC45A1, FOXN3, CPED1, RAPGEF6, SLMO2, AIMP1, GOLGA8G, PRKCE, TYMSOS, CXCR2, TIA1, CADM2, SPICE1, MPP2, ZMYM5, NFAT5, ZBTB18, ZNF600, TMEM30B, FCRL2, STRN3, SPRYD7, PPARGC1B, FBXO11, FZD4, SNCA, SCAF8, HOXB4, PKDREJ, ASH1L, PEX2, C6orf62, AGPAT4, COQ7, KLRG1, OR12D3, SLC31A1, TRAPPC2, USP30, C6orf195, EIF4E3, AC093510.2, CNTN1, PREX2, MAP3K2, ADAM9, INSIG2, ATP6V1B2, A1BG, RTCB, SAMD9, CDK6, CSRNP3, HYDIN, FOXA1, NDC1, SMG1, TMED7-TICAM2, MESDC2, RAB3IP, CASP3, ANKRD20A4, SNX30, CTDNEP1, DSG1, C12orf49, VWA5A, FAT3, FOXO4, YY1, NRK, VPS13A, FAM78B, INPP5A, ZNF236, ETV1, SKAP2, GBP1, VTI1B, MYRFL, RNF144B, JAK1, SLC6A15, SOX5, AMFR, ADRA2B, AC010547.9, CHL1, ATE1, ELOVL6, ZNF678, GREM1, PABPC1L2B, CPA4, TPM3, FOXR2, SMCHD1, SST, LGSN, EXT1, RNF219, ZNF207, ITGA1, BCL11A, ABI2, NTRK2, PEAR1, GRK5, LGALSL, SAMD8, RGPD1, ALCAM, RBM48, ANKRD62, KLHL13, TRPA1, SLC35B3, TEX15, TCF20, TMEM194A, WNK1, ZNF674, HTT, TMEM101, ISOC1, PSMA2, TET2, NOVA1, C12orf29, KLHL5, LSM8, ZNF566, CACNA1E, NOX4, RPS29, CLMN, TANK, ADAMTS12, EOMES, PGRMC2, FDX1, TFRC, CLDN14, SGOL2, ANKRD17, C10orf128, PRDM1, STRADB, OTP, ZC3H8, USP3, NAF1, NOV, FAM196A, MC1R, EIF5, GRIK2, GFOD1, CLHC1, NPM1, CLOCK, GABRA2, IL1F10, ZBTB7C, GNAI3, KIAA1107, ZNF260, BTBD1, NA, MPP1, EFHC2, C2orf43, CTSK, LDB2, EYA4, CD69, USP9Y, PTGFR, SIKE1, POP1, SLC30A8, MTF2, TBC1D5, GPR37, CEP44, SLC9A2, CD109, COPS7B, CNNM2, TRIML2, ARR3, ZFP82, FAM199X |
| miR-122-5p | BAI2, MYB, GYS1, MUC17, NPEPPS, CUX1, SLC17A1, P4HA1, TBR1, HNRNPU, NOL4L, SLC25A34, BRPF1, KIF5B, GNG13, NXNL2, SOX11, AF127577.1, LMNB2, TBC1D10B, IMMT, ADAMDEC1, OCLN, BEST1, GALNTL6, NPIPB5, VIL1, ERV3-1, STAMBP, EBLN2, CTDNEP1, STMN2, SOX6, TSG101, SMURF2, DUSP4, TMX3, FUNDC2, GALNT12, CANX, ZNF169, KDM3B, MECOM, HMGA2, GABRR1, BICD1, EPO, NFAT5, ATF6, MAPRE1, CCNG1, AP1AR, PRR23D2, ZNF827, PRR23D1, TTC6, NPAS3, RFXAP, PELI1, CD40LG, KIAA1244, PTPRG, ADAM10, TBC1D22B, SLC9A1, GIT1, ZNF213, LRR1, HIATL2, GRHL2, AKAP10, SIRPB1, SEC22C, RP11-744N12.3, GALC, WDR47, RP11-826N14.2, VAMP3, CCDC150, ALDOA, ST6GALNAC4, BEND5, FPGT-TNNI3K, PIP4K2A, BPY2, SRSF7, NICN1, PIGS, CLIC4, GPIHBP1, BPY2C, GYPA, BPY2B, PLXNA2, MIXL1, METTL9, CCDC186, AC016586.1, ALG3, TMEM78, MBNL1, MYO9A, AKT3, AC025278.1, RP11-297N6.4, ARHGAP32, NPC1L1, FOXP2, ANKIB1, MRPS11, C8orf86, TRMT61B, SLC7A1, CCNYL1, ECE2, CACNA1C, PKM, RP11-93B14.6, NTRK2, DYRK1A, BATF2, COLEC12, ARHGAP31, PEG10, RHOT1, SPOCK2, TMEM216, SERF1A, CLIC1, AL645730.2, VAMP4, GPLD1, BHLHE41, RBM43, USP8, RP11-1026M7.2, NF1, FAM227B, MED1, SIX3, SRF, DUSP28, C21orf90, TMEM253, EOGT, FOXK2, LNPEP, KIR3DL1, DENND2C, C22orf39, ERICH3, ZNF614, PIEZO2, MOSPD1, KCTD7, LRP10, PFKFB2, RIMS1, DFFB, NEGR1, CLDN2, KIR2DL1, KCTD14, ENY2, LAMC1, NPHS1, BMP1, KIR2DL4, PAPPA, AC092067.1, SLITRK3, ARHGAP19, POU3F3, C19orf54, ERCC1, FOXO3, TMCO5A, CD320, FAM117B, PURB, EFHC1, NDRG3, MICU3, NOS1, FAM102A, ADAM33, STAG3L3, PDZD8, UPK2, ZNF724P, PGS1, WARS2, LTBP2, AGTPBP1, PRKRA, HPSE2, APPL2, PPTC7, SERP1, AMER1, TMED8, CDH6, CACNA2D2, AMD1, ago-01, STXBP4, ARHGEF35, PROK2, TECTB, MSL3, GP2, KCNJ16, DDR2, DSG2, CTCFL, GIMAP6, ENKUR, FBXL5, CWC27, GPD1L, PAK3, UGGT1, TPPP2, TNRC6A, SAMD12, IKZF2, ZDHHC24, CACNA1E, PRPF38B, RPS15A, DSTYK, DCAF4L1, KLHDC1, SLC36A4, CBX6, HOXC8, HIF3A, RDH8, MRC1, C8orf31, RIMBP2, GATAD2B, SPTLC3, FMNL3, SH2D1B, MRC1L1, HOXB9, HECTD3, ZDHHC20, ASCL1, FAM115C, DOCK3, DHX38, FGD2, OCRL, RGS6, GPATCH2, PTPRB, SLC41A1, ADAMTS2, MICB, SCN3B, OSBP2, SCFD2, MPV17, TRIM29, TBCK, SP2, SOAT1, KMT2C, IL18BP, IGFBP5, UBE2L3, LCORL, CD84, RALGAPB, LRP11, GRAMD3, HIF1AN, ZNF331, SLMAP, HHIP, IHH, GREB1L, IGSF5, CA10, GNPDA1, RB1CC1, HEBP1, PLEKHB2, ZNF264, RASGEF1A, ZNF280D, PAX8, RBL1, RDH12, MARCH3, C12orf42, ZCCHC3, ZNF589, GPM6B, GGPS1, AFF2, RPS6KC1, KRTAP2-1, BCR, GLB1L, DLG2, LPAR6, SUMF2, ZSWIM1, GALNT3, PTPN1, NA, SFXN1, TMEM213, USP42, PPARGC1B, ERN2, PTBP3, GIMAP2, INTS8, AL079342.1, ZKSCAN1, NKAIN2, MTF2, SLC25A53, PPT1, LRRD1, RBM5, RRAGC, DDX42, BAIAP2, RNFT2, NUMBL, KIR2DL3, HOXA13, C15orf53, KIAA1751, TSPAN7, AC009113.1, AL513013.1, DNAJC18, DDX10, TOB2, PLCB3, KIAA1324, CECR6, RSPO1, RP11-108K14.4, LILRB4, SUGP2, GAB3, RNF213, CINP, ZNF101, PBOV1, MMP7, IQGAP1, SLC13A5, JADE2, C7orf13, SMG1, PAQR9, ROR1, KCTD12, DAND5, ATP1A2, KLHL20, TEP1, FAM186A, MYO1E, AC009237.1, MEF2D, ISX, SECTM1, CXCR2, LRRTM2, ZNF532, PCDH17, KIF11, DNAJB7, IFNGR2, PLEKHG4B, TRPS1, HR, TFDP2, RUNDC1, RWDD4, ADSS, KIAA0100, KCNC2, GPR156, KCNQ4, KIR3DL2, MYOZ3, GPX3, SEC14L6, MOB3B, NRM, GPR107, UBA52, C15orf57, THG1L, KIAA0368, STK32A, SP100, PTGR1, SLC25A19, MAP3K3, CPM, ZNF124, CDA, ORAI2, FLVCR1, GCNT4, TSPAN2, ADAMTS15, SORCS2, DSCAM, NAB2, ZKSCAN8, DCTN1, TMEM47, RNF165, OLA1, XKR6, CREB1, TMTC2, SEMA4A, CD83, HSPA1L, STX16, PLXNC1, PRMT2, IL1RN, KAT6B, B3GALNT1, FOXJ3, ENAM, CCDC97, NUDT10, PGM2L1, BCL2A1, CALCOCO1, RUNX2, RAVER1, FBXL2, ACSS2, MSRB3, DEDD, STX1B, SFT2D1, BRWD1, BPHL, IGF1R, SGSH, FAM153C, SMYD4, SERF1B, BACH2, KIAA1456, ZNF430, SLC12A1, RABL2B, KMT2A, METTL17, MAPK9, CS, RAB43. |
| miR-125a | NBPF10, ZSWIM5, PCTP, SEMA4D, KNSTRN, KIAA1522, SMEK1, MYT1, FUT4, TNRC6A, LFNG, STARD13, ENPP1, TSNARE1, MFHAS1, DRAM2, NPL, SUV39H1, ENPEP, ZSWIM6, TRIM71, ZNF543, FAM131B, C6orf47, RBM20, GJC3, SLC46A3, FAM169B, NUP210, NCR3LG1, ZSCAN29, MAP3K11, USP2, ARID3B, CRB2, SLC35A4, FBXW4, ABHD6, ORC2, USP6, FAM27C, KLF13, BAK1, MCL1, GCNT1, ESRRA, LBH, LRFN2, TGOLN2, KCNK10, WARS, DUS1L, PDZD3, RFX5, CGN, GTF3C3, GTPBP2, GGA2, C19orf38, SLITRK6, SMG1, JMJD1C, SLC39A9, SLC4A10, MAP3K13, LIN28A, SSTR3, PSTPIP2, DAAM1, UBN1, FAM134A, SEMA4F, EVA1A, TBC1D1, TMTC2, ZFYVE1, OSBPL9, CORO2A, CPSF6, MASP1, CCNJ, TRIAP1, TTPA, ATP10D, PRTG, LRRC10B, INO80D, SBNO1, CCNF, IRF4, KLC2, NBPF20, ZNF704, FPGT-TNNI3K, CYP24A1, SYVN1, NBPF16, C11orf57, DYRK2, SLC35G1, CTTNBP2, VNN3, DDB2, KIAA1841, ORC5, MINK1, ZFP62, VAX1, MAP4K2, NEU1, SLC25A15, ZNF792, PPAT, AIFM1, INTS7, IL16, CHTF8, TAF12, AC009237.1, FAM27A, CDC37L1, VPS4B, RORA, SAMD10, SEMA4C, ZNF827, KCNS3, UBE2D3, TRPS1, SCARB1, ADPRH, TOR2A, ZNF281, CYTH1, ANKRD50, JADE2, TLL1, KLHL6, MRPL30, BRMS1, GANC, KLHL31, TNFAIP3, MLF2, HCN3, SLC7A1, IER3IP1, SH3TC2, CARM1, CSPP1, IST1, IGF2BP3, PTH1R, TMPRSS13, SECISBP2, RABEP2, TET2, MEGF8, BIN2, CD34, DHX33, TRMT5, RAPGEFL1, ZNF460, MAP3K10, DIP2A, MYLK, IBA57, PRDM1, FAM205B, FLT3, FPR3, PCSK7, C19orf54, NT5DC1, C17orf59, KCNJ9, FAM118A, SLC17A7, NRXN1, WI2-3658N16.1, GSTM2, ALPK3, TMEM168, TAF9B, CCR5, LCORL, KIF15, GLYR1, GGT7, PKP4, CELF2-AS1, NLRP1, NLRC5, ZNF839, CMTM1, KPNA6, IL6R, DOCK3, TMPRSS4, TAF4B, KCNIP3, NAIF1, ACAD8, TMLHE, C2orf15, C17orf51, RS1, ARID3A, LRP4, DPY30, TLK1, PTPN18, C10orf54, SIM1, ITGA8, ABTB1, SPEG, ST6GALNAC6, XKRX, TRAF6, BRWD1, SIT1, RNF168, RAD51C, PARP8, BDH1, CXCL13, XPO1, NECAB3, KMT2C, MBD1, NBEAL2, C2orf43, SOX30, BRPF1, EPO, ZNF236, SWSAP1, RYBP, PMM2, TNFSF4, TCTA, SCARB2, KHNYN, NR6A1, NBPF9, CDH13, GRB10, KDM7A, TBX2, RASGRF2, ACSS2, ZNF385A, CNTROB, CHD7, BMF, PYHIN1, RMND5A, NBPF12, CSNK2A1, MBOAT2, NBPF14, MFN1, DPH2, THEMIS2, ZNF691, ATL2, CDR2L, ZNF624, RAB3D, NBPF24, VTCN1, RIT1, OLFML2A, MAPKBP1, ARMC7, LCOR, BLZF1, MAP2K7, UBE2R2, TLE3, MCTP2, ZC3H7B, STXBP5L, TMEM101, RREB1, PHACTR3, SLC26A6, DDX5, LIFR, DIRAS1, SH3BP5L, LYVE1, TMEM2, ARSI, ADAMTS14, SERTAD3, AMER2, FRMD5, ST6GAL1, KCNA7, RTN2, MAPK12, ETV3, BBS4, CTU1, SRRM3, ADAM28, MGAT4A, FAM65B, LIN28B, ITGA9, RUSC2, IKZF3, HOMEZ, ZNF774, BCAN, MEGF9, APLP2, ZNF831, GPR153, NBPF15, KCNA1, MXD4, PAFAH1B1, DAZAP2, TMEM194A, TDG, SAMD14, NXF1, RFC5, GPR107, CDK16, LIPA, ELP6, FOXD2, DYNLT3, LNPEP, LRRC69, CDC42BPG, SULT4A1, AC015804.1, EBF4, PODXL, GJC1, SESTD1, ZNF343, FBXL5, SEL1L, FAM107B, KSR2, SMURF1, TRPM1, ERMP1, STAT3, PPM1H, LCLAT1, VPS37C, SOX11, CTD-2021H9.3, PTPN1, GPATCH2L, ELAVL4, USP7, PBLD, DHX30, PRAMEF18, CTNND2, SLFN5, HDDC3, INTS6, CD48, UBE2G1, TAP2, NIPA1, MAPRE2, TMEM120B, BAP1, PRAMEF3, ABCC5, ETS1, KLHL7, AMIGO2, CSNK2A2, PIK3CB, HINFP, AC013269.5, SS18, SCARA5, ASXL3, CCL4L1, BCL2L14, RP4-539M6.19, MS4A3, ZSWIM4, ALG1, C4orf17, RALGPS2, ROBO4, CHRM3, VWA3A, LRRC8B, TP53INP1, BTN2A2, FMNL3, TMEM63A, PSG4, TOMM40, ICOSLG, TSTA3, ULBP1, KCTD15, GLS, HOXB3, ARHGEF2, MSI1, PSMB8, NBPF11, SZRD1, TMEM26, IKZF4, MSRB3, NIPAL4, ABCC2, AGGF1, SUZ12, ATHL1, LAMTOR3, DTX4, RNF217, KCNC3, DNAJA4, FREM1, PGAP3, FLG, PHC2, PARM1, ENTPD4, PRKCZ, MAP3K1, CHAMP1, DICER1, TMEM180, OPHN1, ACSL6, SSX6, CNOT6L, BAZ2A, E2F2, GOLGA5, SATB2, LEPROT, PPME1, NFATC1, C1orf147, PSMD9, FLOT2, WDR1, ECE1, ZBTB7A, SORD, JARID2, BCAT1, RASAL2, CCNJL, RC3H2, RTP1, NEDD9, BNIP2, IRAK1, LPAR4, DUSP28, VDR, ago-03, FAM185A, SETD6, DIS3L, GAB2, CBFB, GUCD1, TBC1D3F, EIF1AD, PEAK1, SCRT2, BRCA1, LURAP1L, NUTM2G, PDXDC1, ZNF780B, C9orf170, STX16-NPEPL1, PROX2, STX18, NFS1, RBAK, BAG4, CENPP, COL4A3, CACNB1, RFX3, BPI, MYO9A, MED20, MYO1E, TMEM132E, HCN4, NCKAP5L, UNC5C, FOXK2, ZNF546, RGS7BP, PPP2CA, KCNC2, KIAA1467, FNDC3B, MTMR6, C15orf53, SEPN1, ADCY1, CREM, CACNB3, PRELID2, CT62, ZBTB37, RP11-1407O15.2, AP2B1, VPS33B, ELOVL4, KIF1B, FAM129B, TBC1D16, ACOT13, LGI2, GRID1, ACSM2A, RFXANK, CCDC126, PI4K2B, ERBB2IP, KCTD21, ZBED1, RUFY4, ACSM2B, TMEM108, RBM24, TMEM161B, ACACB, ITIH6, FZD4, SLC38A9, ANKS6, RAB6B, CST9, CACNA1E, PLAGL1, TP53BP1, FBXL19, RPS6KA1, CD5L, DUSP6, RP4-695O20__B.10, GP6, NFIB, TEX19, ZNRF3, AGXT2, THOC5, LANCL3, SLC4A4, STOX2, HNRNPA2B1, MCTP1, RAB8B, TBC1D3G, OAZ2, HLA-DQA2, AC008394.1, PAXIP1, ST8SIA3, CSRNP1, RP11-766F14.2, HIC2, B9D1, ZNF787, TP73, VASH1, IRF5, RAP1A, SNX27, KCNH7, SCN4A, ABL2, PRAMEF18, H6PD, ANKRD26, C1orf213, USP37, ENTPD1, NCOR2, ZNF80, FHAD1, MAX, ITCH, PCGF6, SHISA6, RASA2, WDR63, CASP2, C2CD4C, SP9, PSME4, TMEM116, RND2, EDN1, CRCP, GEMIN2, ANKRD13B, CDH11, PPP1R12B, MED15, SLC25A39, QSOX2, TYSND1, AC079612.1, C15orf39, C3orf62, STX6, ELOVL6, TEX26, STC1, TARS2, GRHL1, NUDT16, SGPL1, TOX, TMEM135, EIF5A2, AMBRA1, TBC1D25, RNF175, KIAA1598, GJB7, SORT1, SLC35G3, C1orf109, CELSR2, GOPC, FOLR3, TMEM232, HNRNPUL2, CCR7, AC145676.2, PRDM15, NCLN, KIAA2026, SLC6A15, ITGA1, RHOQ, LRIG3, KLHDC4, FRMPD4, CREB3L2, SP140L, DNAH10, KIAA0556, KIAA1644, TTC21A, SP3, DNAH10OS, FOXS1, FBXO45, UGP2, SYT10, FAT4, PPP1R37, DPF2, TIMM17B, SNURF, TBC1D3H, HIVEP2, CCNC, LYZL6, MKNK2, HOXD9, NPHP3-ACAD11, DTWD2, UBR2, CDK19, PTPN14, FGFR2, CSF2RB, PDE8A, KIF18B, DST, EPSTI1, VANGL1, NFAM1, P2RY8, NKPD1, TMEM136, RASL10B, COL27A1, ATXN1L, GCSAM, FCRL5, MAP3K9, TRIM65, TGIF2, C22orf46, TTC30B, BEND6, PPARA, ANK2, DNAL4, PHKA1, PRSS35, C8orf33, PROX1, SH3BP4, MRAP, PNPLA1, ATP10A, DIDO1, SHPRH, C2CD2L, CDC14B, ABLIM3, ZNF148, IRF1, CREB1, UBR7, ZBTB34, SLC22A13, ZNF707, ZNF396, HK2, C4orf19, CALM2, CPLX4, KRT15, TMEM260, BCL2L2, C7orf43, ASB4, FBXO48, ABCB1, RAF1, MAP6, KDM4B, MTFP1, DDX54, RAVER2, LRTOMT, HCAR3, TSPAN12, PKHD1, ARMCX4, NAA15, SERPINB8, SNX32, FAM53C, AGPAT3, DPP9, SCN5A, TCP10, AQPEP, VCPIP1, KIAA1614, TMEM170B, USP46, TMEM50A, PHOX2B, EDEM1, CNNM1, ZSWIM3, FAM174B, CACNB2, HOXD1, HES2, ASTN2, LMLN, RNF144A, SOGA1, MGA, PHF16, IAH1, METTL21A, MAPK7, SH3RF2, CASZ1, MAMDC2, FBXW8, C1orf210, OTUB2, TINF2, LMOD1, SETD7, NTRK3, CNNM4, GPR157, LETM1, DNMT1, SKIV2L, CYTH2, CCDC71L, SLC39A13, IMPAD1, RASSF3, EPOR, SYT2, PARP14, SCN2B, GAB4, NOX5, C12orf74, POU2F1, DENND6A, ZNF304, IL22RA1, GKAP1, PLEKHG5, FMR1, LRRC8A, PIK3R5, KIAA1244, RHOBTB2, HCN1, HYAL4, RP11-121M22.1, SLC8A2, PAPOLB, RNPEPL1, ATP5G2, SNX33, TREML2, ARHGAP40, NICN1, FGF5, CALCOCO2, CPM, LACC1, ZNF75D, CAMSAP3, AMMECR1L, CER1, DUSP7, FKBP15, HIPK2, TMED9, PRR23A, AGPAT4, C1orf110, ATAT1, CNR2, BMPR1B, C10orf105, HNRNPR, THY1, PTCH1, MAVS, FBXO42, LHX9, SNX24, TMEM231, EXO5, CHD8, CDH23, KIRREL, LIF, TRIM14, FOXQ1, DGKH, SGCD, REST, CACNA1B, SLC6A17, ATIC, TUB, MGAT5, FUT1, TAZ, ABCC11, ZCCHC8, KXD1, LONRF2, ARHGAP20, KRTAP5-8, TECPR1, GALNT7, SCML2, SCN4B, ZNF559-ZNF177, ATOH8, CCDC169, CAMK4, NDST1, TMEM72, FAM71F1, ANO6, HNF4G, CNOT3, EPS8L2, MTMR3, C17orf103, PSG8, EHD1, ANO3, NCAM2, COL24A1, BCL2, WIPF2, CD69, ZNF687, PHTF1, PHF20, AGBL4, HIF1AN, HRNR, RIMS3, C17orf102, APPL1, HIATL1, TXNRD1, NWD2, LLGL2, TBC1D20, CBX7, NCAPD3, KMT2D, DRP2, GRAMD2, IFNAR2, APOBEC4, RBM38, PTPRB, SAMD7, TRPV5, ZDHHC15, ZNF362, ago-02, GPR160, ENTPD7, SLC46A1, KIAA0753, CYYR1, ZNF652, CHRM5, PVRL2, SH2D7, KCNB1, C6orf132, HCAR2, UBOX5, MAT2A, HEMK1, ZNF12, RNASEH2C, MARCH7, ANGPT2, TPPP, GLB1L2, EPHA8, C7orf72, OGFR, SLC2A10, SLC2A14, CCDC120, CAPN14, SOCS4, PANK2, UBE2L3, KBTBD13, C6orf223, ACTR8, PDE7B, FOXP2, NT5C3L, C5orf64, QPRT, EDC3, ADPGK, CDK15, ZNF254, APOBEC3B, FAM222B, FAM178B, UQCC1, ATXN1, VEZF1, SLC30A2, VPS37B, LL22NC03-63E9.3, DUSP3, CHRNA2, EEF1G, PSME3, ZNF592, CAB39L, CFLAR, NOL4L, FMO2, KMO, ARHGEF3. |
